# Acute-phase innate immune responses in SIVmac239-infected Mamu-B*08+ Indian rhesus macaques may contribute to the establishment of elite control

**DOI:** 10.3389/fimmu.2024.1478063

**Published:** 2024-10-22

**Authors:** Brandon C. Rosen, Kaitlin Sawatzki, Michael J. Ricciardi, Elise Smith, Inah Golez, Jack T. Mauter, Núria Pedreño-López, Aaron Yrizarry-Medina, Kim L. Weisgrau, Logan J. Vosler, Thomas B. Voigt, Johan J. Louw, Jennifer Tisoncik-Go, Leanne S. Whitmore, Christakis Panayiotou, Noor Ghosh, Jessica R. Furlott, Christopher L. Parks, Ronald C. Desrosiers, Jeffrey D. Lifson, Eva G. Rakasz, David I. Watkins, Michael Gale

**Affiliations:** ^1^ Department of Pathology, George Washington University School of Medicine and Health Sciences, Washington, DC, United States; ^2^ Department of Immunology, Center for Innate Immunity and Immune Disease, School of Medicine, University of Washington, Seattle, WA, United States; ^3^ Wisconsin National Primate Research Center, University of Wisconsin-Madison, Madison, WI, United States; ^4^ International AIDS Vaccine Initiative, New York, NY, United States; ^5^ Department of Pathology, University of Miami Miller School of Medicine, Miami, FL, United States; ^6^ AIDS and Cancer Virus Program, Frederick National Laboratory for Cancer Research, Frederick, MD, United States

**Keywords:** simian immunodeficiency virus (SIV), cytotoxic T lymphocytes (CTLs), human immunodeficiency virus (HIV), vaccines, acquired immunodeficiency syndrome (AIDS)

## Abstract

**Introduction:**

Spontaneous control of chronic-phase HIV/SIV viremia is often associated with the expression of specific MHC class I allotypes. HIV/SIV-specific CD8+ cytotoxic T lymphocytes (CTLs) restricted by these MHC class I allotypes appear to be critical for viremic control. Establishment of the elite controller (EC) phenotype is predictable in SIVmac239-infected Indian rhesus macaques (RMs), with approximately 50% of Mamu-B*08+ RMs and 20% of Mamu-B*17+ RMs becoming ECs. Despite extensive characterization of EC-associated CTLs in HIV/SIV-infected individuals, the precise mechanistic basis of elite control remains unknown. Because EC and non-EC viral load trajectories begin diverging by day 14 post-infection, we hypothesized that hyperacute innate immune responses may contribute to viremic control.

**Methods:**

To gain insight into the immunological factors involved in the determination of EC status, we vaccinated 16 Mamu-B*08+ RMs with Vif and Nef to elicit EC-associated CTLs, then subjected these 16 vaccinees and an additional 16 unvaccinated Mamu-B*08+ controls to repeated intrarectal SIVmac239 challenges. We then performed whole-blood transcriptomic analysis of all 32 SIVmac239-infected Mamu-B*08+ RMs and eight SIVmac239-infected Mamu-B*08 ^–^ RMs during the first 14 days of infection.

**Results:**

Vaccination did not provide protection against acquisition, but peak and setpoint viremia were significantly lower in vaccinees relative to controls. We did not identify any meaningful correlations between vaccine-induced CTL parameters and SIVmac239 acquisition rate or chronic-phase viral loads. Ultimately, 13 of 16 vaccinees (81%) and 7 of 16 controls (44%) became ECs (viremia ≤ 10,000 vRNA copies/mL plasma for ≥ 4 weeks). We identified subsets of immunomodulatory genes differentially expressed (DE) between RM groupings based on vaccination status, EC status, and MHC class I genotype. These DE genes function in multiple innate immune processes, including the complement system, cytokine/chemokine signaling, pattern recognition receptors, and interferon-mediated responses.

**Discussion:**

A striking difference in the kinetics of differential gene expression among our RM groups suggests that Mamu-B*08-associated elite control is characterized by a robust, rapid innate immune response that quickly resolves. These findings indicate that, despite the association between MHC class I genotype and elite control, innate immune factors in hyperacute SIV infection preceding CTL response development may facilitate the establishment of the EC phenotype.

## 1 Introduction

CD8+ cytotoxic T lymphocytes (CTLs) are a critical component of the natural adaptive immune response to HIV/SIV infection and are therefore attractive options for utilization in HIV/SIV prophylaxis and cure strategies. In infected individuals, CTLs mediate the two- to four-log reductions in peak viremia to setpoint levels during acute infection by killing infected CD4^+^ T cells (1–4). Furthermore, rare individuals expressing specific major histocompatibility complex (MHC) class I allotypes can spontaneously control chronic-phase viral replication in the absence of antiretroviral therapy (ART) (5–8). Numerous studies have implicated the HIV/SIV-specific CTLs restricted for these MHC class I allotypes in this phenomenon, known as elite control. We have previously demonstrated that this elite controller (EC) phenotype is reproducible in SIVmac239-infected Indian rhesus macaques (*Macaca mulatta*; RMs) expressing the Mamu-B*08 and Mamu-B*17 allotypes, where approximately 50% and 20% of animals, respectively, become ECs in the absence of any prophylactic or therapeutic interventions (7, 8). These EC frequencies are substantially higher than the EC frequencies for HIV-infected individuals expressing the EC-associated HLA-B*27 and HLA-B*57 MHC class I allotypes (5, 6), and can be enhanced by vaccination (9–12), making the SIVmac239 EC model valuable for studying elite control. However, despite extensive studies of EC immunology in both HIV-infected humans and SIV-infected RMs, the mechanistic basis of elite control remains unclear (9–23). While CTLs restricted for EC-associated MHC class I allotypes are clearly a major factor, the elite control phenomenon cannot be fully explained by the frequencies, phenotypic characteristics, or effector functionalities of these CTLs alone.

We have performed multiple studies of SIVmac239-infected Mamu-B*08+ RMs to better understand the EC phenomenon, with the ultimate objective of developing a novel vaccination strategy that recapitulates the EC phenotype in individuals lacking the EC-associated MHC class I alleles. We first identified Mamu-B*08 as an EC-associated allele by analyzing the SIVmac239 chronic-phase viral loads and MHC class I haplotypes of nearly 200 infected RMs, finding that 38% of all ECs were Mamu-B*08+ and that possession of the Mamu-B*08 allele conferred a sevenfold decrease in chronic-phase viremia (8). Characterization of the SIV-specific CTL repertoires of Mamu-B*08+ RMs led to the identification of immunodominant, Mamu-B*08-restricted Vif- and Nef-derived epitopes (24). Vaccination with these minimal optimal epitopes (Vif RL8, Vif RL9, and Nef RL10) greatly enhanced the incidence of the EC phenotype in Mamu-B*08+ RMs, and higher acute-phase frequencies of Nef RL10-specific CTLs correlated with lower viremia (9). We therefore hypothesized that Nef RL10-specific CTLs were the predominant CTL population responsible for elite control and vaccinated Mamu-B*08+ RMs with this epitope. However, vaccination with Nef RL10 alone did not protect against SIVmac239 acquisition and failed to enhance the incidence of the EC phenotype due to the rapid emergence of escape mutants (10). We then vaccinated Mamu-B*08+ RMs with *vif* and *nef* minigenes spanning the three immunodominant epitopes, as well as a fourth subdominant epitope, Nef RL9b (12). Three of the eight vaccinees never became infected after a total of 10 intrarectal challenges, while all six unvaccinated controls became infected within six challenges. As expected, the five infected vaccinees exhibited a greater propensity for elite control than unvaccinated animals, although two of the infected vaccinees eventually lost control of viremia, likely due to viral escape. While we have definitively demonstrated that vaccine-mediated boosting of certain SIV-specific, Mamu-B*08-restricted CTL populations dramatically enhances the EC phenotype, we have been unable to fully define the mechanistic basis for elite control, in the context of both “enhanced” elite control in vaccinees and “natural” elite control in unvaccinated Mamu-B*08+ RMs.

Thus, our objectives for the present study were the following: 1) to determine whether vaccine-mediated boosting of EC-associated CTLs in Mamu-B*08+ RMs provides any degree of protection against intrarectal SIVmac239 acquisition, and 2) to obtain additional insight into – and perhaps elucidate – the mechanistic basis of elite control in Mamu-B*08+ RMs. Based on the proven immunogenicity of our previous *vif* and *nef* minigene vaccination strategy, we vaccinated 16 Mamu-B*08+ RMs with a heterologous prime-boost-boost regimen of viral vectors encoding these minigenes (12), then subjected these 16 vaccinees and an additional 16 Mamu-B*08+ unvaccinated controls to repeated intrarectal challenges with SIVmac239 once every two weeks. Based on previous observations that the EC phenotype is determined within the first 2-3 weeks post-infection (9, 10, 12), we hypothesized that hyperacute innate immune responses, such as those that facilitate the subsequent expansion of EC-associated CTLs, are a critical factor in the determination of EC status in chronic infection. We therefore performed longitudinal functional genomics analyses of whole blood to evaluate the transcriptomes of all 32 Mamu-B*08+ animals and an additional eight Mamu-B*08^–^ historical controls during the first 14 days of SIVmac239 infection. We directly compared gene expression profiles of 1) unvaccinated ECs and non-ECs, 2) all ECs and non-ECs (both vaccinated and unvaccinated), 3) all vaccinees and unvaccinated controls, and 4) unvaccinated Mamu-B*08+ animals and unvaccinated Mamu-B*08^–^ animals. Notably, such hyperacute-phase studies cannot be performed in HIV-infected humans, since it is very difficult to determine the precise date of HIV infection and obtain patient samples at such early post-infection timepoints.

Herein, we report the immunogenicity and efficacy of our vaccination regimen, the elite control outcomes for all RMs infected with SIVmac239 in this study, correlation analyses of immunologic and virologic parameters, and transcriptomic characterization of our unique cohort of elite control-predisposed RMs during acute SIVmac239 infection. We discuss the implications of our findings in the contexts of HIV/SIV vaccine development and further efforts to more fully elucidate the mechanistic basis for elite control of HIV/SIV viral replication.

## 2 Materials and methods

### 2.1 Research animals and ethics statement

A total of 40 Indian rhesus macaques (RMs; *Macaca mulatta*) were used in this study (**Table 1**). All RMs were housed at the Wisconsin National Primate Research Center (WNPRC) at the University of Wisconsin-Madison. MHC class I genotyping was performed by the WNPRC by PCR with sequence-specific primers or by deep sequencing of exon amplicons (25, 26). Animal care and welfare protocols were compliant with the guidelines described in the Weatherall report (27) and the National Research Council’s *Guide for the Use and Care of Laboratory Animals* (28). All experimental procedures were performed according to protocols approved by the University of Wisconsin Graduate School Animal Care and Use Committee. Protocols describing animal caretaking procedures (e.g. food, housing, enrichment activities, standard medical care) and efforts to minimize animal suffering (e.g. anesthesia, euthanasia) can be found in the “Research animals and ethics statement” sections of our previous vaccination studies also performed at the WNPRC (10–12). Animals were anesthetized for vaccinations and blood draws via intramuscular ketamine administration (dosage 5-12 mg/kg). As in our previous studies, animals exhibiting signs or symptoms of SIV-induced disease (e.g. opportunistic infections) were euthanized to minimize potential suffering. Animals were euthanized by first administering general anesthesia (ketamine ≥ 15 g/kg intramuscularly), then administering an IV overdose of sodium pentobarbital (≥ 50 mg/kg).

**Table 1:** Animal characteristics.

Experimental Group	Animal ID	MHC Class I Alleles^a^	Age (Years)^b^	Sex
Mamu-B*08+ Vaccinees	r08014	Mamu-A*01, Mamu-A*02, Mamu-B*08	13.6	M
	r14065	Mamu-B*08	8.1	F
	r16007	Mamu-B*08	6.1	F
	r17017	Mamu-A*01, Mamu-B*08	4.8	M
	r17115	Mamu-B*08	4.4	M
	rh2932	Mamu-B*08	6.4	F
	rh2943	Mamu-B*08	7.1	F
	rh3006	Mamu-A*02, Mamu-B*08	5.3	F
	rh3011	Mamu-A*01, Mamu-B*08	4.6	F
	rh3032	Mamu-B*08	4.9	M
	rh3033	Mamu-A*02, Mamu-B*08	4.9	M
	rh3036	Mamu-B*08	4.8	F
	rh3037	Mamu-B*08	4.9	F
	rh3045	Mamu-B*08	5.2	M
	rh3048	Mamu-B*08	4.9	F
	rh3053	Mamu-B*08	5.3	M
Mamu-B*08+ Unvaccinated Controls	r12048	Mamu-B*08	9.6	M
	r14010	Mamu-B*08	8.1	M
	r14095	Mamu-B*08	7.4	F
	r15057	Mamu-B*08	6.9	M
	r15058	Mamu-A*02, Mamu-B*08	6.3	M
	r15093	Mamu-B*08	5.8	F
	r16003	Mamu-B*08	6.0	F
	r17039	Mamu-B*08	4.7	F
	r17082	Mamu-B*08	5.1	F
	r17100	Mamu-B*08	4.8	M
	r17103	Mamu-B*08	4.2	M
	r18066	Mamu-B*08	4.2	F
	r18067	Mamu-B*08	3.4	F
	rh2494	Mamu-B*08	16.2	M
	rh2504	Mamu-A*01, Mamu-B*08	14.1	M
	rh2527	Mamu-B*08	16.7	F
Mamu-B*08^–^ Unvaccinated Controls	r05035	Mamu-A*02	16.0	F
	rh2756	Mamu-A*01	17.0	F
	rh2810	Mamu-A*01	12.1	F
	rh2848	Mamu-A*02	7.6	M
	rh2861		9.5	M
	rh2995	Mamu-A*01	4.6	M
	rh3000	Mamu-A*02	4.4	M
	rh3013	Mamu-A*02	4.4	F

a MHC class I alleles relevant for spontaneous control of SIVmac239 viremia and/or characterization of SIVmac239-specific CTL populations.

b Age at time of infecting SIVmac239 challenge.

### 2.2 Vaccinations

Sixteen Mamu-B*08+ RMs were vaccinated with a heterologous prime-boost-boost regimen of recombinant viral vectors encoding the SIVmac239 Vif and Nef proteins. Vaccine doses were spaced eight weeks apart, and the final vaccine dose was administered approximately 22 weeks prior to the first SIVmac239 challenge. RMs were first vaccinated with two recombinant adenovirus type 5 (rAd5) vectors encoding *vif* and *nef* minigenes (12, 29). Each animal received a total dosage of 10^11^ particles of rAd5-*vif* and 1.4 x 10^11^ particles of rAd5-*nef*, administered intramuscularly to both shoulders as a cocktail. Eight weeks later, RMs were vaccinated with a recombinant vesicular stomatitis virus (rVSV) vector encoding a Nef-Tat-Vif fusion protein (12). Each animal received 10^7^ particles of the rVSV vector administered intramuscularly to the thigh. Eight weeks following the rVSV vaccination, RMs were vaccinated with a cocktail of three recombinant rhesus rhadinovirus (rRRV) vectors encoding a Rev-Tat-Nef fusion protein, Nef alone, and Vif alone (12, 30, 31). Each animal received 7.05 x 10^7^ genome equivalents of each rRRV vector intravenously and 2.1 x 10^8^ genome equivalents of each rRRV vector intrarectally. An additional sixteen Mamu-B*08+ RMs were enrolled in this study to serve as unvaccinated controls. While empty viral vectors alone can theoretically elicit innate immune responses, we elected to forego vaccination of our controls with empty vectors due to cost limitations and previous experience with empty viral vectors in multiple SIVmac239 vaccination trials, in which we observed no effect on SIVmac239 acquisition rates or control of viral replication after challenge (9, 11, 12).

### 2.3 SIVmac239 challenges and viral load quantification

RMs were challenged with SIVmac239 intrarectally once every two weeks, starting at week 38 post-rAd5 vaccination (22 weeks after the final rRRV vaccination dose). Once an animal became infected, as defined by detectable viremia at day 7 and/or day 10 post-challenge, it did not receive additional challenges. We established an intrarectal marginal infectious dose of 10 TCID_50_ for our clonal, rhesus PBMC-passaged SIVmac239 challenge stock by challenging eight Mamu-B*08^–^ RMs intrarectally with different dosages (mean challenges to infection = 3.5). For the 32 Mamu-B*08+ RMs, we first performed 12 challenges at the 10 TCID_50_ dosage, resulting in the infection of 9/16 vaccinees and 12/16 unvaccinated controls. The next seven challenges were performed at 50 TCID_50_, infecting all remaining animals except one control. This final animal was infected on the 20th challenge at a 250 TCID_50_ dose. Plasma viral loads were measured using a Gag-targeted quantitative RT-PCR assay, essentially as previously described (32).

### 2.4 Sample processing and cryopreservation

PBMCs and plasma were isolated from EDTA-anticoagulated blood by Ficoll-Paque Plus (Cytiva) density centrifugation. Following density gradient centrifugation, PBMCs were washed in R10 medium (RPMI 1640 with GlutaMAX, 10% heat-inactivated FBS, antimycotic-antibiotic). In some cases, red blood cells were lysed by resuspending PBMCs in ACK lysis buffer (Gibco) and incubating for 5 minutes at room temperature. After an additional wash in R10 medium, PBMCs were enumerated, pelleted by centrifugation, and resuspended in cryopreservation medium (67.5% RPMI 1640 with GlutaMAX, 22.5% heat-inactivated FBS, 10% DMSO). PBMCs were cooled to -80°C at a controlled rate, then transferred to liquid nitrogen freezers for long-term storage. For the flow cytometry-based immunological assays in this study, cryopreserved PBMCs were thawed in a 37°C water bath, washed twice in R10 medium, then recounted. For RNA preservation for transcriptomic analyses, whole blood was collected in PAXgene^®^ Blood RNA Tubes (BD Biosciences), stored initially at -20°C for 24 hours, then moved to -80°C for long-term storage.

### 2.5 pMHCI tetramer staining and CTL phenotypic characterization

pMHCI tetramer staining and CTL phenotypic characterization by flow cytometry was performed essentially as previously described (33). Briefly, PBMCs (approximately 2.0 x 10^6^ per tube) were stained with BV421- or PE-conjugated pMHCI tetramers corresponding to four immunodominant SIVmac239-specific, Mamu-B*08-restricted CTL populations (NIH Tetramer Core Facility, Emory University, Atlanta, GA) for 30 minutes at room temperature. Cells were stained for an additional 30 minutes with a cocktail containing the following fluorophore-conjugated monoclonal antibodies (mAbs) and amine-reactive viability dye: anti-CD4 BV605 (clone OKT4, BioLegend), anti-CD8 BV785 (clone RPA-T8, BioLegend), anti-CD14 APC (clone M5E2, BioLegend), anti-CD20 APC (clone 2H7, BioLegend), anti-CD159a APC (clone Z199, Beckman Coulter), anti-CD28 PE-Cy7 (clone CD28.2, BioLegend), anti-CCR7 FITC (clone 150503, BD Biosciences), and Far Red live/dead dye (Invitrogen). Cells were washed in 1% FBS in PBS, fixed with Cytofix (BD Biosciences) for 20 minutes at 4°C, then permeabilized by washing in Perm/Wash Buffer (BD Biosciences). PBMCs were stained intracellularly with anti-CD3 PerCP-Cy5.5 (clone SP34-2, BD Biosciences), anti-Ki-67 PE-CF594 (clone B56, BD Biosciences), and anti-granzyme B Alexa Fluor 700 (clone GB11, BD Biosciences) for 30 minutes at 4°C, then washed in Perm/Wash Buffer prior to acquisition. Samples were acquired on a BD LSR II flow cytometer. Analysis was performed using FlowJo software. Flow cytometry gating strategy is shown in **Supplementary Figure S1**. Tetramer frequencies correspond to the percentage of tetramer^+^ cells among live CD3^+^ CD8^+^ CD4^–^ CD14^–^ CD20^–^ CD159a^–^ lymphocytes. Terminally differentiated effector memory (T_EM2_) CTLs were further gated as CD28^–^ CCR7^–^.

### 2.6 CD107a degranulation assay with intracellular cytokine staining

CD107a/ICS assays were performed essentially as previously described (33). Briefly, PBMCs in R10 medium (2 x 10^6^ per tube) were incubated with anti-CD28 and anti-CD49d costimulatory mAbs (clones L293 and 9F10, respectively; BD Biosciences), anti-CD107a PE (clone H4A3, BioLegend), and various peptide stimuli for one hour at 37°C. Minimal optimal peptides (synthesized by GenScript Biotech Corp., Piscataway, NJ) were used at a final concentration of 5 μM. SIVmac239 Vif and Nef peptide pools (NIH HIV Reagent Program) containing 15mers overlapping by 11 amino acids were used at a final concentration of 5 μg/mL per peptide. Following addition of brefeldin A (BioLegend) and GolgiStop (BD Biosciences) to each sample, cells were incubated for an additional eight hours at 37°C then stored at 4°C overnight until staining the following day. Cells were washed once in 1% FBS in PBS, then stained with a cocktail containing the following fluorophore-conjugated mAbs and amine-reactive viability dye: anti-CD4 BV605 (clone OKT4, BioLegend), anti-CD8 BV785 (clone RPA-T8, BioLegend), anti-CD14 APC (clone M5E2, BioLegend), anti-CD20 APC (clone 2H7, BioLegend), anti-CD159a APC (clone Z199, Beckman Coulter), and Far Red live/dead dye (Invitrogen). Cells were washed, then fixed and permeabilized using Cytofix (BD Biosciences) and Perm/Wash buffer (BD Biosciences). Cells were stained intracellularly for 30 minutes at 4°C with the following cocktail of fluorophore-conjugated mAbs: anti-CD3 PerCP-Cy5.5 (clone SP34-2, BD Biosciences), anti-CD69 PE-Cy7 (clone FN50, BioLegend), anti-TNF-α APC-Cy7 (clone MAb11, BioLegend), and anti-IFN-γ BV421 (clone B27, BioLegend). Following a final wash in Perm/Wash buffer, samples were acquired on a BD LSR II flow cytometer. Analysis was performed using FlowJo software. Flow cytometry gating strategy is shown in **Supplementary Figure S2**. The total frequency of responding CTLs was defined as live CD3^+^ CD8^+^ CD69^+^ CD4^–^ CD14^–^ CD20^–^ CD159a^–^ lymphocytes staining positive for CD107a *or* TNF-α *or* IFN-γ. Detectable responses were defined by *or* gate frequencies at least two-fold greater than the *or* gate frequency for unstimulated PBMCs from the same animal. Polyfunctionality was defined as the percentage of live CD3^+^ CD8^+^ CD69^+^ CD4^–^ CD14^–^ CD20^–^ CD159a^–^ lymphocytes staining positive for CD107a *and* TNF-α *and* IFN-γ simultaneously. All possible effector function combinations among CD69^+^ CTLs were analyzed using the Boolean gating function in FlowJo.

### 2.7 RNA isolation and sequencing

RNA was isolated using the RNAdvance Blood Kit (Beckman) following the manufacturer’s instructions. RNA quality was assessed using the 4200 TapeStation System (Agilent). mRNAseq libraries were constructed using KAPA RNA HyperPrep Kit with RiboErase (HMR) Globin in conjunction with the KAPA mRNA Capture Kit (Roche Sequencing). Equimolar amounts of sample were multiplexed and library quality was evaluated using the 4200 TapeStation instrument prior to loading onto the Illumina NovaSeq 6000 instrument at the Northwest Genomics Center (University of Washington) for PE100 cycles. Samples were demultiplexed using bcl2fastq (Illumina). Adapters and low-quality ends were trimmed from FASTQ files with Trim Galore (v0.6.10) and quality analysis performed by FastQC (v0.11.2). Reads mapping to ribosomal RNA and globin were removed using Bowtie2 (v2.5.1), with an index composed of human, mouse and rat rRNA sequences, resulting in at least 25 million reads per sample. Remaining sequences were aligned to the RM genome (Mmul_10.109) using STAR (v2.7.10b). Alignment results showed > 90% of reads mapped to the RM genome.

### 2.8 Differential gene expression analyses

Statistical processing and analysis of RNAseq count data was performed in the R statistical computing environment (R Core Team 2023). Gene counts were filtered by a row mean of 3 or greater and then normalized using edgeR with calcNormFactors using TMM normalization. Counts were log2 transformed using limma with voom. Principal Component Analysis was performed to inspect global transcription data for sources of variation and sex was identified as a covariate for model inclusion. Differential expression (DE) analysis was performed for each time point against baseline samples per animal using linear model fit per gene with limma. Significance cut-offs were set at log fold change > 1.5 and adjusted *p*-value < 0.05 calculated using Benjamini-Hochberg false discovery rate correction. GO enrichment analysis for biological processes was performed on significantly up- and down-regulated DE genes using Fisher’s exact test with false discovery rate (FDR) calculation (34).

## 3 Results

### 3.1 Vaccination elicits elite control-associated CTLs in Mamu-B*08+ RMs

**Figure 1:**
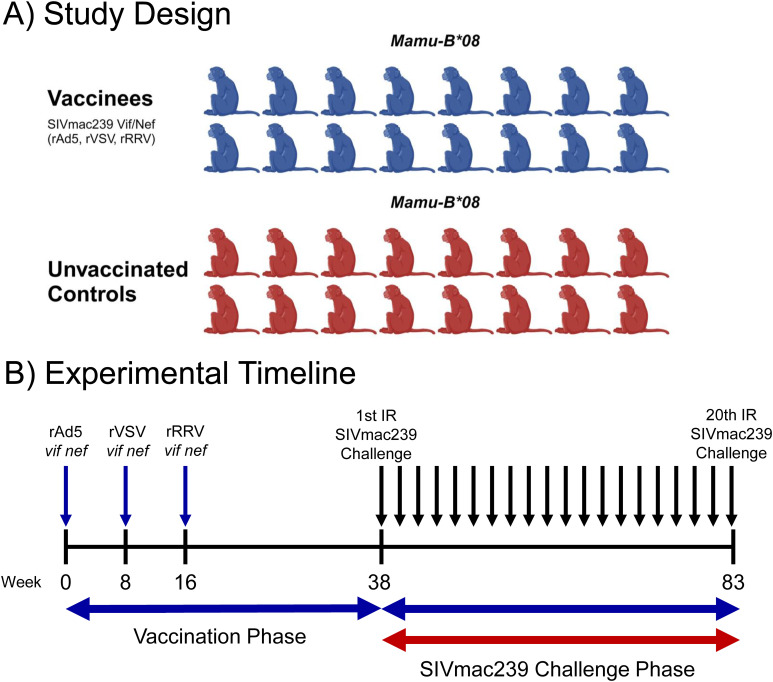
Study design and experimental timeline. Thirty-two Mamu-B*08+ RMs were recruited for this study. Animals were distributed into vaccinated (n = 16) and unvaccinated (n = 16) groups of equal size **(A)**. One group was vaccinated with a heterologous prime-boost-boost regimen of different viral vectors encoding *vif* and *nef* minigenes as shown in **(B)**. Both groups were then subjected to intrarectal challenges with a marginal infectious dose of SIVmac239 (10 TCID_50_ for challenges 1-12, 50 TCID_50_ for challenges 13-19, and 250 TCID_50_ for challenge 20) once every two weeks. Once an animal became infected, it no longer received additional intrarectal challenges, meaning that most animals received fewer than the maximum 20 challenges. The graphic in panel A was created using BioRender.com.

Thirty-two Mamu-B*08+ RMs were included in this study (**Table 1**). Sixteen animals were vaccinated with heterologous viral vectors encoding SIVmac239 *vif* and *nef* minigenes spanning the immunodominant Mamu-B*08-restricted Vif RL8, Vif RL9, and Nef RL10 epitopes. RMs were first vaccinated with recombinant adenovirus type 5 (rAd5) vectors, followed by vaccination with a recombinant vesicular stomatitis virus (rVSV) vector eight weeks later, and recombinant rhesus rhadinovirus (rRRV) vectors after another eight weeks (**Figure 1**).

**Figure 2:**
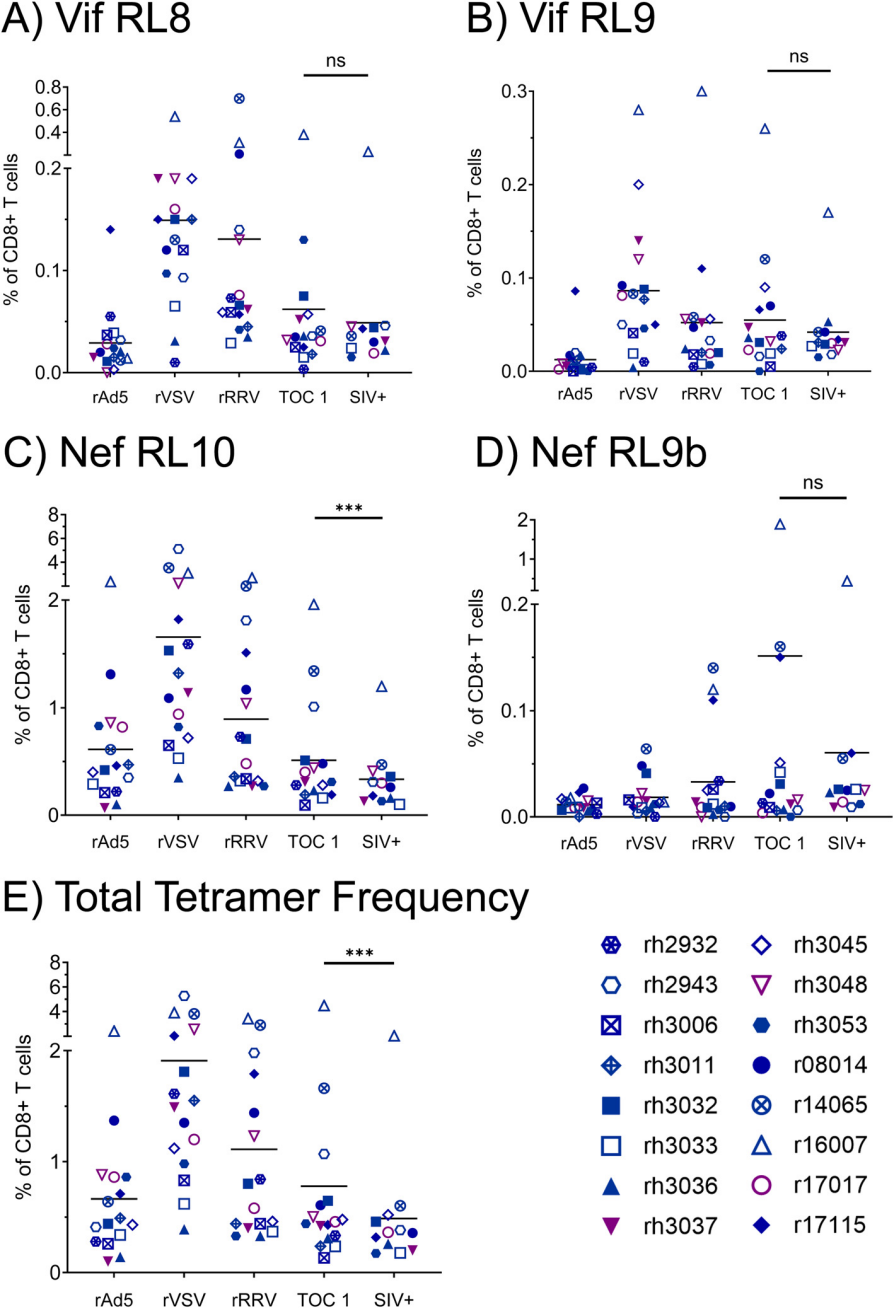
Frequencies of vaccine-induced, Mamu-B*08-restricted CTLs specific for immunodominant Vif- and Nef-derived CTL epitopes during the vaccination phase. Vaccinee PBMCs were stained with fluorophore-conjugated Mamu-B*08 tetramers presenting the indicated Vif- and Nef-derived CTL epitopes **(A–D)** at the following timepoints: two weeks post-rAd5 vaccination (“rAd5”), two weeks post-rVSV vaccination (“rVSV”), two weeks post-rRRV vaccination (“rRRV”), at the time of the first SIVmac239 challenge (“TOC 1”), and at the time of the infecting challenge for each animal (“SIV+”). Plots depict frequencies of tetramer^+^ cells among all CD8^+^ T cells (defined as live CD3^+^ CD8^+^ CD4^–^ CD14^–^ CD20^–^ CD159a^–^ lymphocytes). **(E)** Summative frequencies of the four tetramer^+^ CTL populations [shown individually in **(A–D)**] for each vaccinee. Statistical significance testing was performed to assess differences in tetramer frequencies across all timepoints (Friedman test) and to determine whether CTL responses waned during the challenge phase (Wilcoxon test, comparing the “TOC 1” and “SIV+” timepoints). Friedman test *P*-values were < 0.0001, 0.0006, < 0.0001, 0.0737, and < 0.0001 for panels **(A–E)** respectively. Wilcoxon test results are shown in each panel: *P < 0.05, **P < 0.01, ***P < 0.001, ****P < 0.0001, ns, not significant.

We assessed vaccine immunogenicity by flow cytometry-based analyses of SIV-specific CTL populations within longitudinal PBMC samples from all 16 vaccinees. We performed pMHCI tetramer staining with CTL phenotypic characterization at timepoints two weeks after each vaccination dose, as well as at the time of the first SIVmac239 challenge and at the time of the infecting SIVmac239 challenge for each animal (**Figure 2**). CTLs specific for at least one of the four tetramers, corresponding to the three immunodominant Mamu-B*08-restricted epitopes Vif RL8, Vif RL9, and Nef RL10, and the subdominant Mamu-B*08-restricted Nef RL9b epitope, were detected in all 16 vaccinees, and most animals possessed multiple tetramer^+^ CTL populations. Tetramer frequencies were generally highest following the rVSV boost and declined during the 22-week interval between the rRRV boost and the first intrarectal SIV challenge. Notably, tetramer frequencies continued to decline throughout the SIV challenge phase. At all timepoints tested, frequencies of Nef RL10-specific CTLs were far greater than frequencies of the three other Mamu-B*08-restricted CTL populations analyzed.

**Figure 3:**
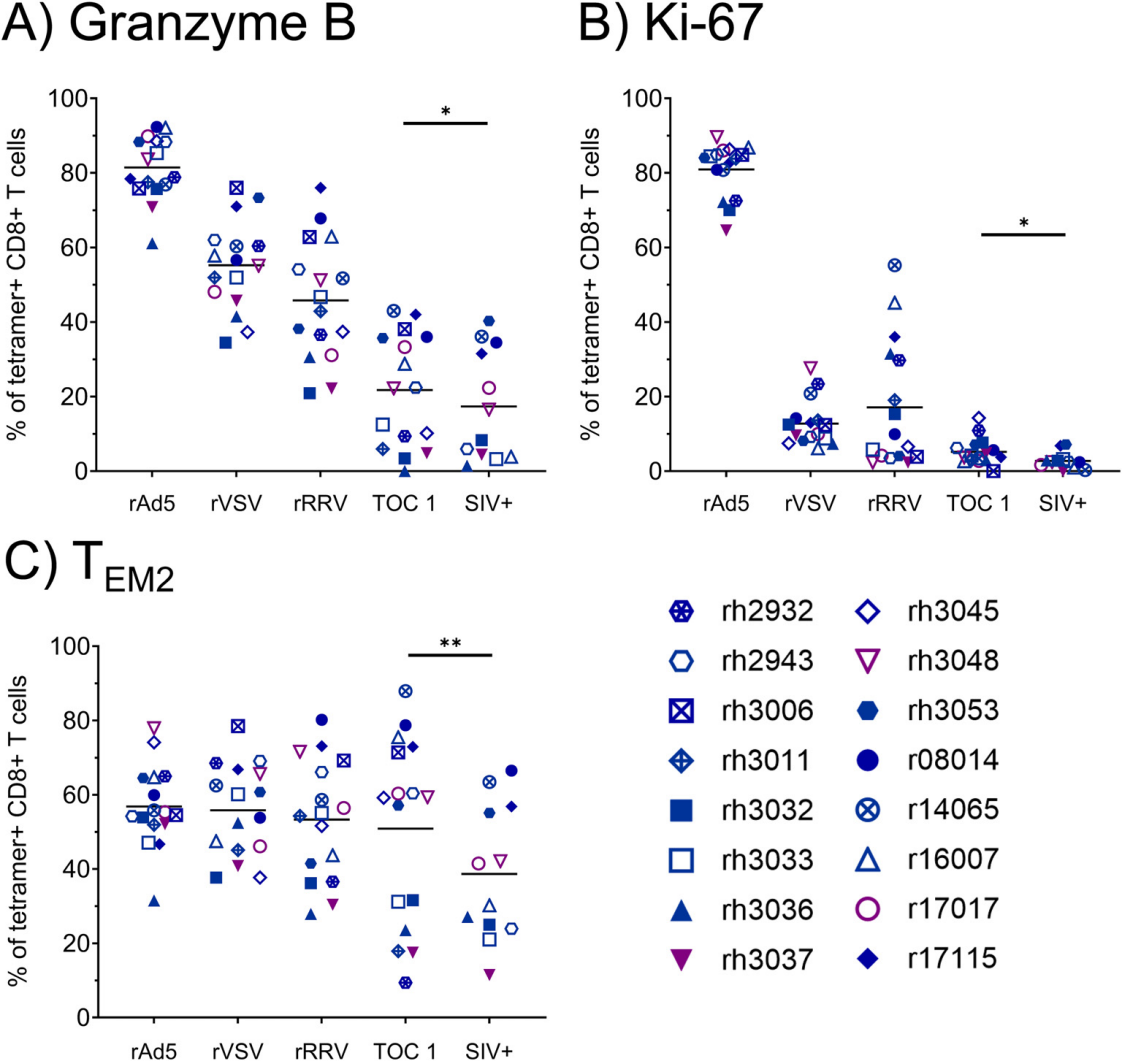
Phenotypic characterization of vaccine-induced Nef RL10-specific CTLs during the vaccination phase. Vaccinee PBMCs were stained with fluorophore-conjugated Nef RL10-Mamu-B*08 tetramers and fluorophore-conjugated mAbs to assess the following CTL phenotypic characteristics: **(A)** frequencies of granzyme B^+^ CTLs, **(B)** frequencies of Ki-67^+^ CTLs, and **(C)** frequencies of terminally-differentiated effector memory (T_EM2_) CTLs, defined as CD28^–^ CCR7^–^ CTLs. PBMCs from the following timepoints were analyzed: two weeks post-rAd5 vaccination (“rAd5”), two weeks post-rVSV vaccination (“rVSV”), two weeks post-rRRV vaccination (“rRRV”), at the time of the first SIVmac239 challenge (“TOC 1”), and retrospectively at the time of the infecting challenge for a given animal (“SIV+”). Plots depict frequencies of Nef RL10-Mamu-B*08 tetramer^+^ CD8^+^ T cells (defined as live tetramer^+^ CD3^+^ CD8^+^ CD4^–^ CD14^–^ CD20^–^ CD159a^–^ lymphocytes) meeting the staining criteria for each phenotypic characteristic. Statistical significance testing was performed to assess differences in Nef RL10-specific CTL phenotypic characteristics across all timepoints (Friedman test) and to determine whether these phenotypic characteristics changed during the challenge phase (Wilcoxon test, comparing the “TOC 1” and “SIV+” timepoints). Friedman test *P*-values were < 0.0001, < 0.0001, and 0.02073 for panels **(A-C)** respectively. Wilcoxon test results are shown in each panel: *P < 0.05, **P < 0.01.

Due to the low frequencies of CTLs specific for Vif RL8, Vif RL9, and Nef RL9b, we focused on Nef RL10-specific CTLs for phenotypic analyses. The rAd5 prime elicited Nef RL10-specific CTLs with high proliferative and cytotoxic potential, as indicated by high levels of Ki-67 and granzyme B expression (**Figure 3**). However, expression of both markers appeared to decline with each vaccination dose, and only 21.7 ± 15.1% of Nef RL10-specific CTLs stained positive for granzyme B at the time of the first SIV challenge. Frequencies of terminally differentiated effector memory (T_EM2_) Nef RL10-specific CTLs remained relatively constant throughout the vaccination phase (between 50.9 ± 25.1% and 56.8 ± 11.1%) but declined in some animals during the SIV challenge phase (to 38.7 ± 18.3% at the time of the infecting SIV challenge).

**Figure 4:**
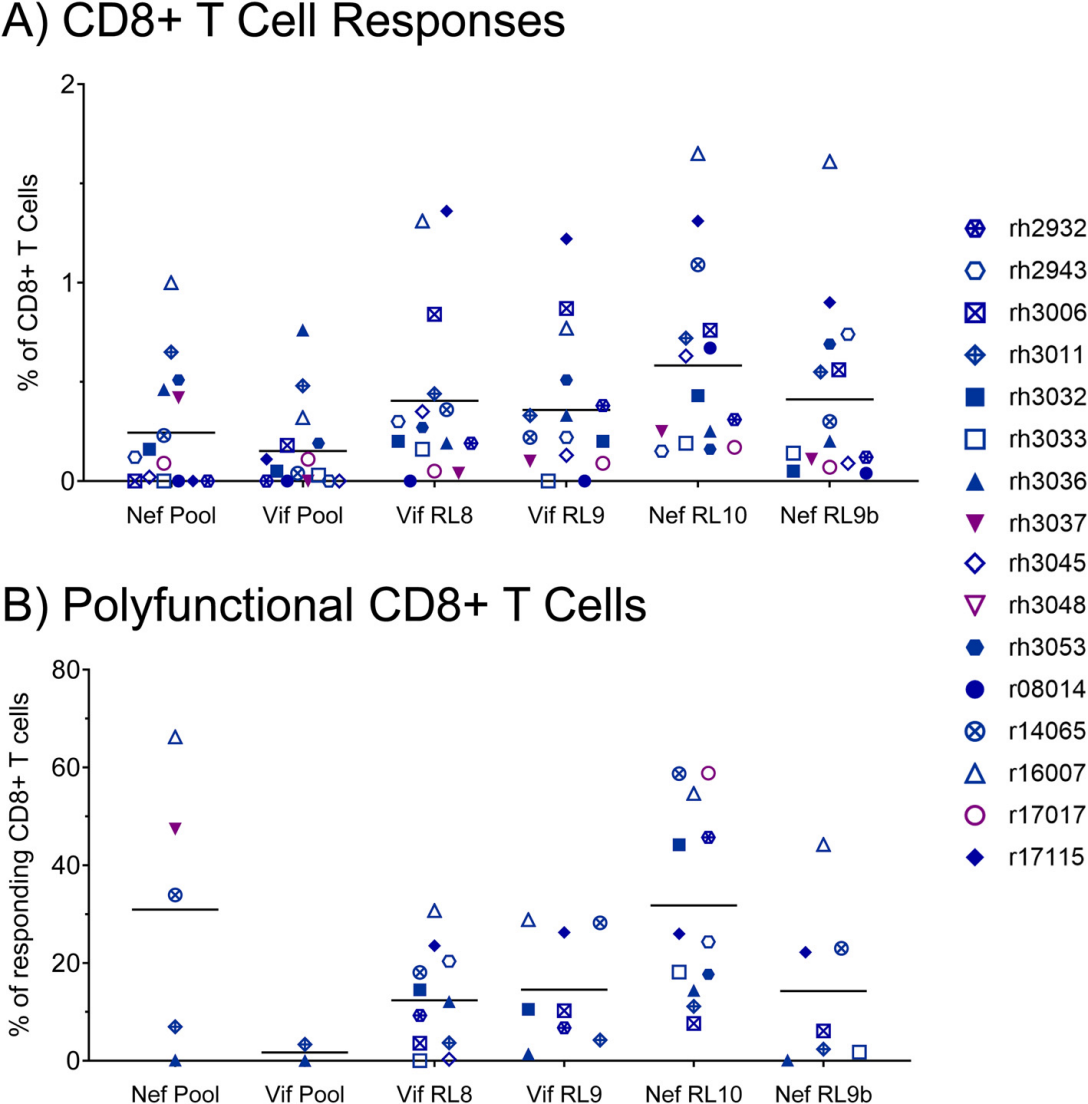
Frequencies of total responding and polyfunctional Vif- and Nef-specific CTLs at the time of the first SIVmac239 challenge. The antigen responsiveness and effector function profiles of Vif- and Nef-specific CTLs within vaccinee PBMCs were assayed by CD107a degranulation assay with intracellular cytokine staining (CD107a/ICS) at the time of the first SIVmac239 challenge. **(A)** Frequencies of CD8^+^ T cells mounting responses to the indicated peptide stimuli; **(B)** frequencies of polyfunctional CD8^+^ T cells among all CD8^+^ T cells responding to each stimulus. Responding CD8^+^ T cells were defined as CD69^+^ CD3^+^ CD8^+^ CD4^–^ CD14^–^ CD20^–^ CD159a^–^ lymphocytes staining positive for CD107a, IFN-γ, or TNF-α. Polyfunctional CD8^+^ T cells were defined as CD69^+^ CD3^+^ CD8^+^ CD4^–^ CD14^–^ CD20^–^ CD159a^–^ lymphocytes staining positive for all three effector function markers (CD107a, IFN-γ, and TNF-α) simultaneously.

To assess the effector function profiles of the vaccine-induced, SIV-specific CTLs in our vaccinees, we performed CD107a degranulation assays with intracellular cytokine staining (CD107a/ICS) at the time of the first SIV challenge. PBMCs were stimulated with Vif or Nef peptide pools (15mers overlapping by 11 amino acids spanning the entire proteins) or one of the four minimal optimal peptides corresponding to the immunodominant or subdominant Mamu-B*08-restricted CTL populations. CTL responses to the Vif and Nef peptide pools were detectable in 2/16 and 5/16 vaccinees at the time of the first SIV challenge, with mean response frequencies of 0.24% and 0.15% of CD8+ T cells within PBMCs, respectively (**Figure 4A**). CTL response frequencies were considerably larger for the Mamu-B*08-restricted minimal optimal epitopes (between 0.36% and 0.58% on average), with 11/16 vaccinees mounting detectable responses to both Vif RL8 and Nef RL10. We further characterized effector function profiles for CTLs responding to the various peptide stimuli. CTLs specific for the Nef peptide pool and Nef RL10 epitope alone exhibited the highest degree of polyfunctionality (30.9 ± 27.6% and 31.8 ± 19.3%, respectively), defined as CD69 expression with simultaneous degranulation (CD107a^+^), IFN-γ expression, and TNF-α expression (**Figure 4B**). Further analysis of effector function repertoires of CTLs revealed that the majority of CTLs responding to Vif RL8 (52%) and Vif RL9 (54%) were only capable of degranulation, but not cytokine production (**Supplementary Figures S3A, B**). CTLs responding to Nef RL10 exhibited greater cytokine production capabilities, as 33% of responding CTLs were polyfunctional (CD107a^+^ IFN-γ^+^ TNF-α^+^), while 36% of CTLs were capable of degranulation alone (**Supplementary Figure S3C**).

### 3.2 Vaccine-mediated elicitation of elite control-associated CTLs in Mamu-B*08+ RMs does not protect against intrarectal SIVmac239 acquisition

Twenty-two weeks after the final vaccination dose (rRRV boost), we began intrarectal SIVmac239 challenges of the 16 vaccinated Mamu-B*08+ RMs and an additional 16 unvaccinated Mamu-B*08+ RMs. Animals were challenged intrarectally on a biweekly basis with a marginal infectious dose (10 TCID_50_, equivalent to 1.07 x 10^6^ vRNA copies) of a clonal, RM PBMC-passaged SIVmac239 stock. This challenge stock was titrated in eight RMs lacking the elite control-associated Mamu-B*08 and Mamu-B*17 MHC class I alleles, and a 10 TCID_50_ challenge dose established infection within 3.5 challenges, on average (**Supplementary Figure S4**). An infecting challenge was defined by detectable plasma SIV viral loads (limit of quantification = 15 vRNA copies/mL plasma) at day 7 and/or day 10 post-challenge; only animals with viral loads < 15 vRNA copies/mL plasma on day 7 and day 10 viral loads were re-challenged.

**Figure 5:**
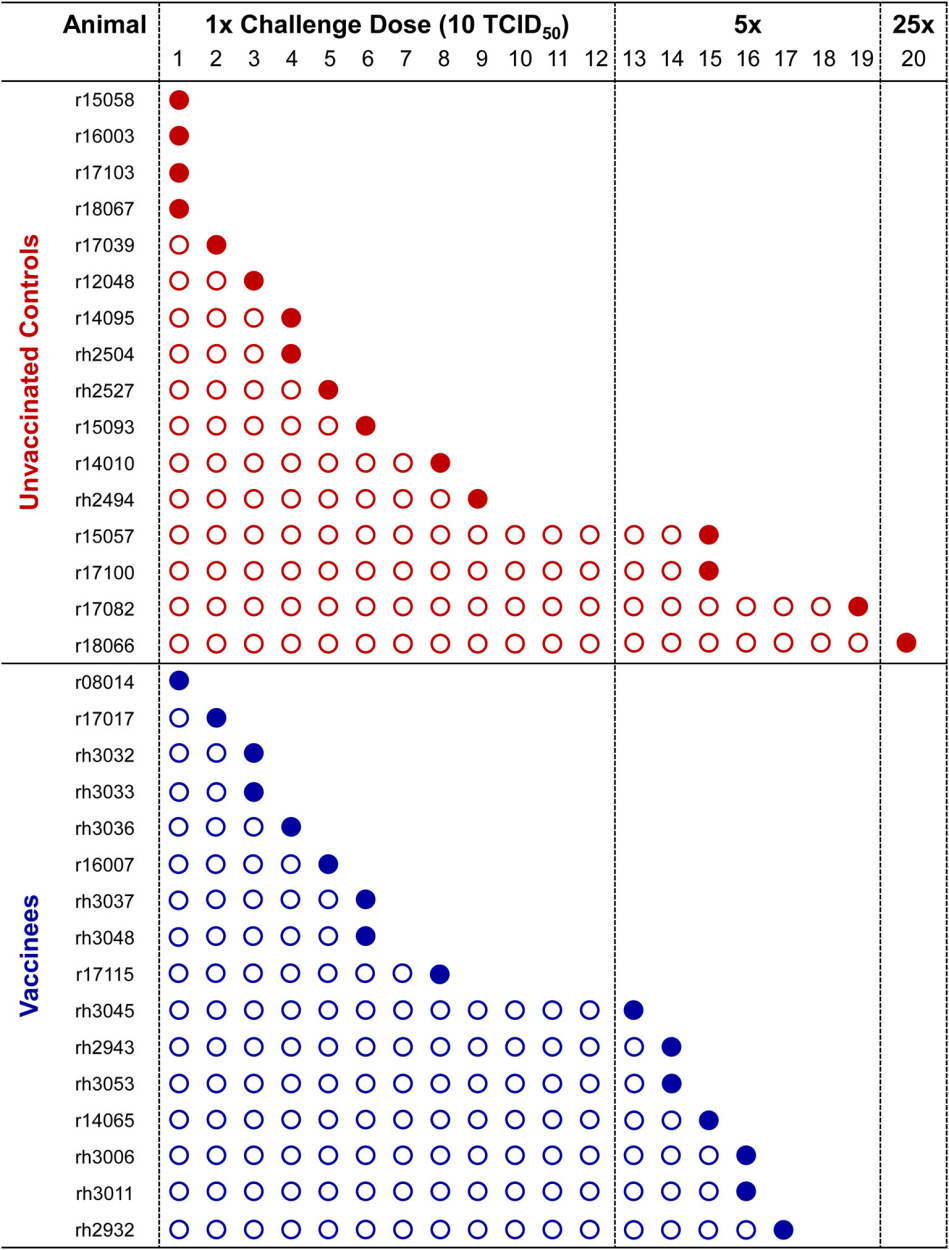
Kinetics of SIVmac239 acquisition in vaccinated and unvaccinated Mamu-B*08+ RMs. All 32 Mamu-B*08+ RMs were subjected to intrarectal challenges with a marginal infectious dose of SIVmac239 approximately once every two weeks. Infecting and noninfecting challenges are indicated by filled and open circles, respectively. Establishment of SIVmac239 infection was defined by detectable SIV plasma viral loads at day 7 and/or day 10 post-challenge, and animals meeting these criteria were not re-challenged. The intrarectal challenge dose was increased fivefold to 50 TCID_50_ on the 13th challenge, then again increased fivefold to 250 TCID_50_ on the 20th challenge.

**Figure 6:**
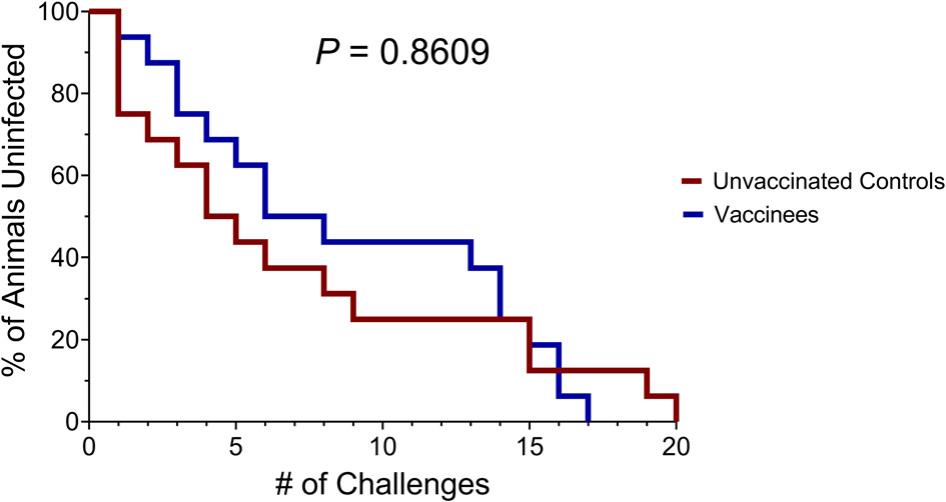
Survival analysis of SIVmac239 acquisition in challenged vaccinated and unvaccinated Mamu-B*08+ RMs. The *P*-value of 0.8609 was computed using the log-rank/Mantel-Cox test.

For the first twelve challenges, unvaccinated controls and vaccinees became infected at roughly equal rates, except for the first challenge, where four controls and only one vaccinee became infected (**Figure 5**). By the ninth challenge, 21/32 animals had become infected (12/16 controls and 9/16 vaccinees). However, none of the remaining animals were infected following three subsequent challenges, prompting us to increase the challenge dose fivefold to 50 TCID_50_ (5.35 x 10^6^ vRNA copies) for the 13th challenge. All vaccinees had become infected by the 17th challenge and 15/16 unvaccinated animals had become infected by the 19th challenge. On the 20th challenge, we again increased the challenge dose fivefold to 250 TCID_50_ (2.68 x 10^7^ vRNA copies) and successfully infected the final unvaccinated animal. Despite the apparent resistance of some RMs to intrarectal SIVmac239 acquisition, we did not identify any statistically significant associations between SIVmac239 acquisition rate and animal attributes such as weight, sex, or age (data not shown). Comparison of SIV acquisition rates between vaccinees and unvaccinated controls via survival curve analysis (log-rank/Mantel-Cox test) yielded a nonsignificant *P*-value of 0.8609 (**Figure 6**). Therefore, our vaccination regimen did not provide protection against intrarectal SIVmac239 acquisition in these Mamu-B*08+ RMs with a genetic predisposition for elite control.

Despite the lack of vaccine efficacy in preventing SIVmac239 acquisition, we performed correlation analyses to identify possible associations between the frequencies or phenotypes of vaccine-induced CTLs and SIVmac239 acquisition rate. We only found statistically significant positive correlations between the frequencies of CTLs responding to the Vif RL8 and Vif RL9 peptides (by CD107a/ICS assay) at the time of the first SIVmac239 challenge and the number of challenges required to infect a vaccinee (**Table 2**). However, the frequencies of CTLs specific for the Vif RL8- and Vif RL9-Mamu-B*08 tetramers at the time of the first challenge did not correlate with SIVmac239 acquisition rate.

**Table 2:** Correlations between immunologic parameters and SIVmac239 acquisition^a^ in vaccinated Mamu-B*08+ RMs.

Immunologic Parameter^b^	n	Correlation Coefficient (Spearman r)	*P-*value
Vif RL8 tetramer frequency	16	-0.2186	0.4122
Vif RL9 tetramer frequency	16	-0.1357	0.6140
Nef RL10 tetramer frequency	16	-0.2506	0.3459
Nef RL9b tetramer frequency	16	-0.1578	0.5567
Total tetramer frequency (Vif RL8 + Vif RL9 + Nef RL10 + Nef RL9b)	16	-0.2389	0.3700
T_EM2_ frequency, Nef RL10-specific CTLs	16	-0.1490	0.5793
Granzyme B+ frequency, Nef RL10-specific CTLs	16	0.1283	0.6336
Frequency of responding CTLs (Vif peptide pool + Nef peptide pool)	15	0.0860	0.7584
Frequency of responding CTLs (Vif RL8)	15	0.5246	**0.0468**
Frequency of responding CTLs (Vif RL9)	15	0.5959	**0.0211**
Frequency of responding CTLs (Nef RL10)	15	0.3008	0.2738
Frequency of responding CTLs (Nef RL9b)	15	-0.2384	0.4075
Frequency of polyfunctional CTLs (Nef peptide pool)	5	0.0000	0.9999
Frequency of polyfunctional CTLs (Vif RL8)	11	-0.1050	0.7584
Frequency of polyfunctional CTLs (Vif RL9)	8	-0.2156	0.6100
Frequency of polyfunctional CTLs (Nef RL10)	12	-0.3163	0.3139
Frequency of polyfunctional CTLs (Nef RL9b)	7	0.3063	0.5008

a SIVmac239 acquisition rate was defined as the number of intrarectal challenges required to infect a given animal for purposes of this correlation analysis.

b Immunologic parameters at the time of the first SIVmac239 challenge (week 38 post-rAd5 vif/nef vaccination).

*P*-values in bold are statistically significant (*P* < 0.05).

### 3.3 Vaccinated Mamu-B*08+ RMs have a greater predisposition for elite control than unvaccinated Mamu-B*08+ RMs

**Figure 7:**
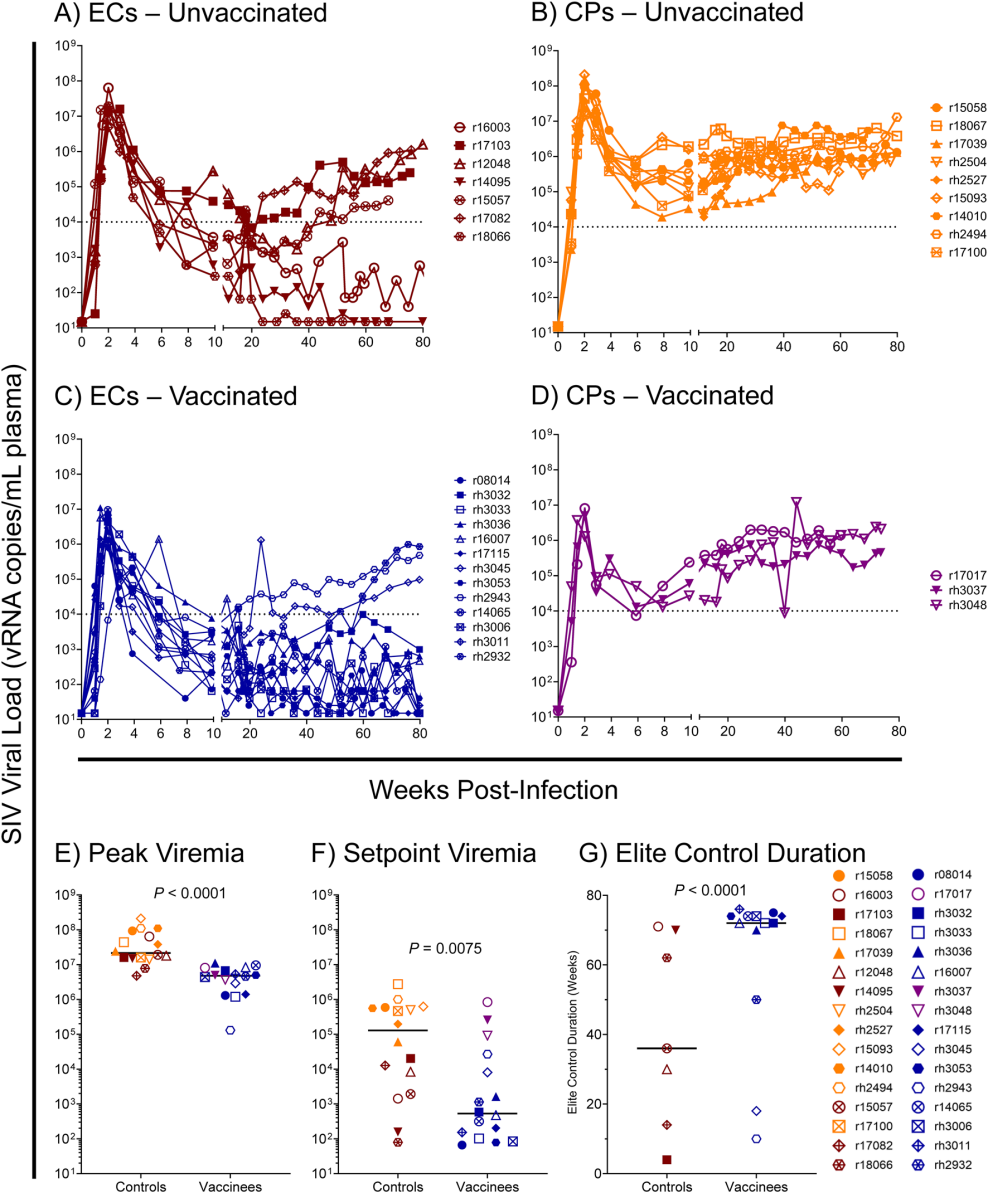
SIVmac239 viral loads in vaccinated and unvaccinated Mamu-B*08+ RMs. Longitudinal SIVmac239 plasma viral loads for all Mamu-B*08+ RMs in this study. Animals are grouped based on vaccination status and chronic-phase viremia status (elite controller [EC] or common progressor [CP]): **(A)** unvaccinated ECs, **(B)** unvaccinated CPs, **(C)** vaccinated ECs, and **(D)** vaccinated CPs. Comparison of **(E)** peak viremia and **(F)** setpoint viremia in vaccinated and unvaccinated Mamu-B*08+ RMs by the Mann-Whitney test. Peak viremia was defined as the highest viral load measurement at any timepoint; setpoint viremia was defined as the geometric mean of all viral load measurements between weeks 10 and 40 post-infection. **(G)** Comparison of the duration of elite control in vaccinated and unvaccinated Mamu-B*08+ RMs classified as ECs by the Mann-Whitney test. Elite control duration was defined as the number of consecutive weeks an animal maintained viral loads ≤ 10,000 vRNA copies/mL plasma.

Based on our previous studies of SIVmac239-infected Mamu-B*08+ RMs, we expected approximately 50% of animals to spontaneously control viremia and become ECs without vaccination, and we expected an even larger proportion of vaccinees to become ECs. For this study, we defined elite control as chronic-phase viremia of less than or equal to 10,000 vRNA copies/mL plasma, maintained for a minimum of four consecutive weeks. While chronic-phase viremia of 10,000 copies/mL is typical of untreated HIV-infected humans, it is low for SIVmac239-infected RMs due to the high pathogenicity of the SIVmac239 clone. Chronic-phase viremia in non-ECs – both Mamu-B*08+ or Mamu-B*08^–^ – is routinely around 10^6^ vRNA copies/mL plasma (**Figure 7** and **Supplementary Figure S4**). Ultimately, 13 of 16 vaccinees (81%) and 7 of 16 unvaccinated controls (44%) became ECs based on this criterion, and both peak and setpoint viremia were significantly lower in vaccinees than in unvaccinated controls (**Figure 7**, **Supplementary Figures S5**, **S6**). Additionally, the duration of elite control – that is, the period of time during which viral loads remained ≤ 10,000 vRNA copies/mL plasma – was significantly longer in vaccinees than in unvaccinated controls (**Figure 7G**). In our third cohort of eight unvaccinated Mamu-B*08^–^ RMs used for technical controls, all animals experienced the high levels of chronic-phase viremia characteristic of common progressors (CPs), as expected for animals lacking elite control-associated MHC class I alleles (**Supplementary Figure S4**). Despite the greater elite control predisposition of Mamu-B*08+ vaccinees relative to unvaccinated Mamu-B*08+ RMs, there were no statistically significant immunological correlates of elite control when comparing setpoint viremia and the quantity and quality of peripheral blood vaccine-induced CTL responses (**Table 3**).

**Table 3:** Correlations between immunologic parameters and setpoint SIVmac239 viral loads^a^ in vaccinated Mamu-B*08+ RMs.

Immunologic Parameter^b^	n	Correlation Coefficient (Spearman r)	*P-*value
Vif RL8 tetramer frequency	16	0.1311	0.6263
Vif RL9 tetramer frequency	16	0.1235	0.6485
Nef RL10 tetramer frequency	16	0.2771	0.2965
Nef RL9b tetramer frequency	16	-0.1206	0.6564
Total tetramer frequency (Vif RL8 + Vif RL9 + Nef RL10 + Nef RL9b)	16	0.2176	0.4168
T_EM2_ frequency, Nef RL10-specific CTLs	16	-0.2676	0.3152
Granzyme B+ frequency, Nef RL10-specific CTLs	16	-0.4294	0.0986
Frequency of responding CTLs (Vif peptide pool + Nef peptide pool)	15	0.0876	0.7556
Frequency of responding CTLs (Vif RL8)	15	-0.2143	0.4421
Frequency of responding CTLs (Vif RL9)	15	-0.2181	0.4318
Frequency of responding CTLs (Nef RL10)	15	-0.1893	0.4983
Frequency of responding CTLs (Nef RL9b)	15	-0.0590	0.8350
Frequency of polyfunctional CTLs (Nef peptide pool)	5	0.2000	0.7833
Frequency of polyfunctional CTLs (Vif RL8)	11	0.2636	0.4348
Frequency of polyfunctional CTLs (Vif RL9)	8	-0.2143	0.6191
Frequency of polyfunctional CTLs (Nef RL10)	12	0.5455	0.0708
Frequency of polyfunctional CTLs (Nef RL9b)	7	0.1429	0.7825

a Setpoint SIVmac239 viral load was defined as the geometric mean viral load of all timepoints between weeks 10 and 40 post-infection.

b Immunologic parameters at the time of the first SIVmac239 challenge (week 38 post-rAd5 vif/nef vaccination).

### 3.4 Differential expression of immunomodulatory genes during acute SIV infection distinguishes elite controllers from common progressors in vaccinated, unvaccinated, and Mamu-B*08^–^ RMs

**Figure 8:**
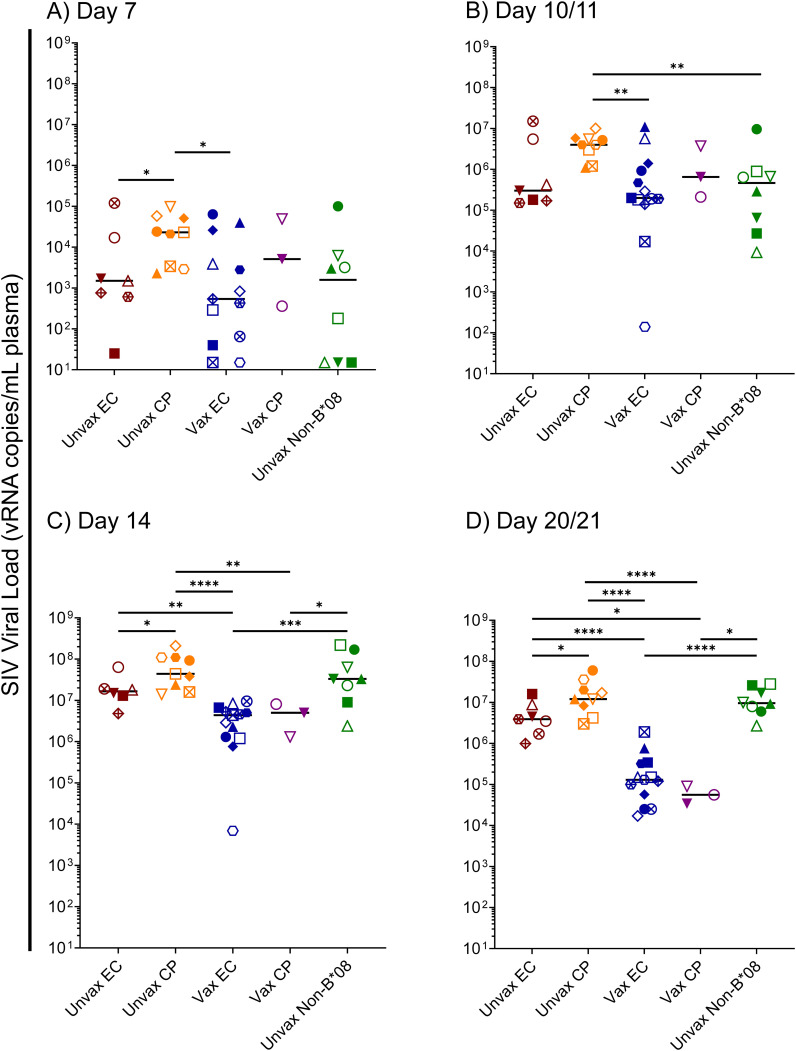
Kinetics of acute-phase SIVmac239 viremia in Mamu-B*08+ RMs and Mamu-B*08^–^ RMs. Comparison of SIVmac239 plasma viral loads in various Mamu-B*08+ RM groupings (unvaccinated EC, unvaccinated CP, vaccinated EC, vaccinated CP) and unvaccinated Mamu-B*08^–^ RMs at the indicated acute-phase timepoints: **(A)** day 7, **(B)** day 10/11, **(C)** day 14, and **(D)** day 21. Statistically significant differences in viremia were identified using the Mann-Whitney test (*P < 0.05, **P < 0.01, ***P < 0.001, ****P < 0.0001). All other pairwise group comparisons did not yield statistically significant *P*-values. Possible multiple testing error was assessed by the Kruskal-Wallis test; *P*-values were 0.1046, 0.0385, < 0.0001, and < 0.0001 for the day 7, day 10/11, day 14, and day 21 timepoints, respectively, indicating that the differences observed for the day 7 timepoint are likely not statistically significant.

To further understand the factors involved in the determination of EC status in Mamu-B*08+ RMs, we focused upon the first 14 days of SIVmac239 infection in our 40-animal cohort – comprised of 16 vaccinated Mamu-B*08+ RMs, 16 unvaccinated Mamu-B*08+ RMs, and eight unvaccinated Mamu-B*08^–^ RMs. Median viral loads of ECs and CPs began to diverge as early as day 10/11 post-infection, and by day 14, median viral loads of vaccinees were significantly lower than those of unvaccinated controls (**Figure 8**). By day 21 post-infection, median viral loads in vaccinees were at least 100-fold lower than those of unvaccinated CPs and unvaccinated Mamu-B*08^–^ RMs. Unvaccinated Mamu-B*08+ RMs that became ECs had intermediate viral loads, about 30-fold higher than vaccinees. Based on these analyses of acute-phase viremia in the context of the eventual EC/CP status of each animal, we hypothesized that acute-phase immune responses – perhaps even those preceding the development of efficacious adaptive immune responses – contributed to the determination of eventual EC/CP status.

**Figure 9:**
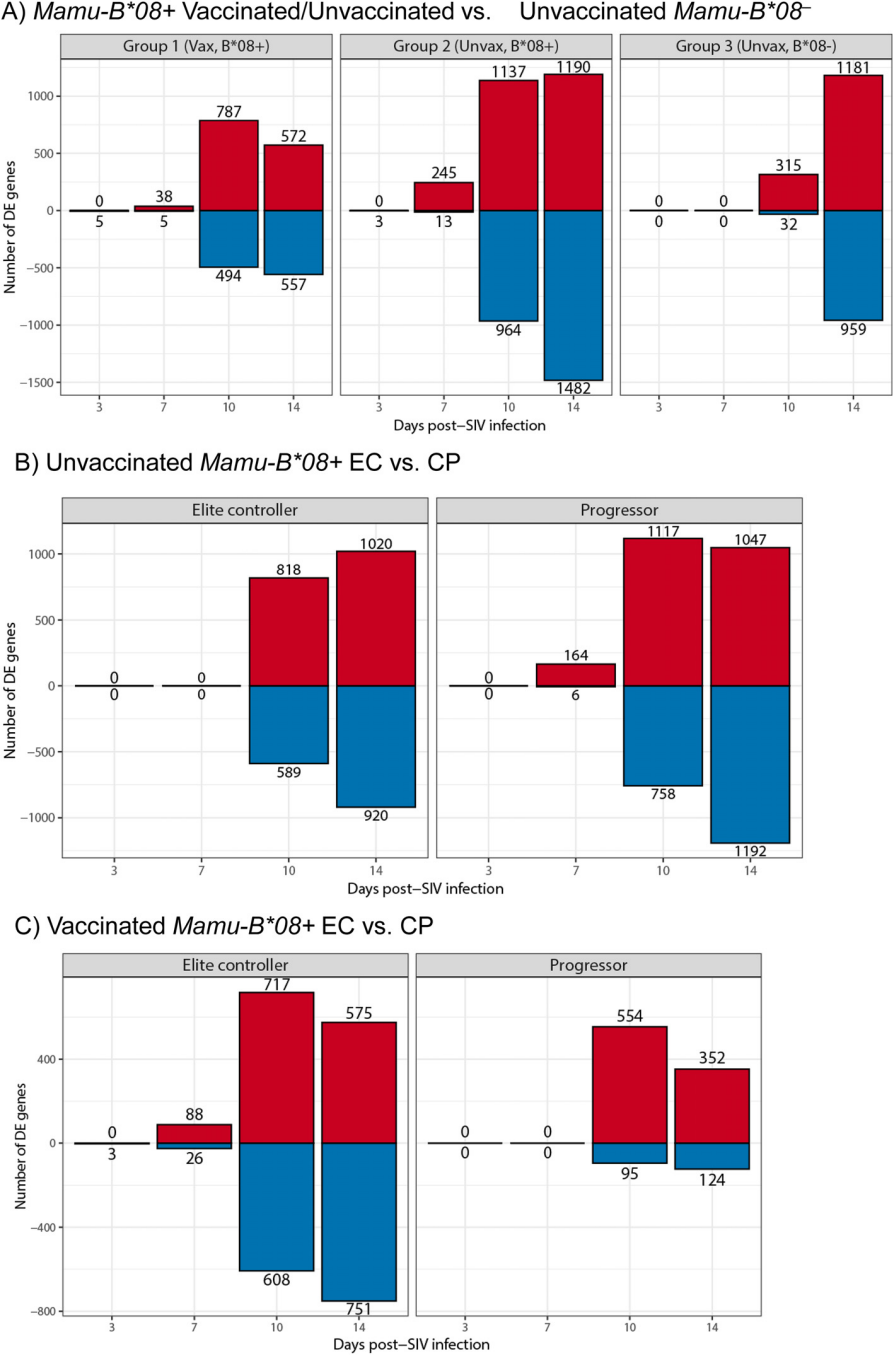
Differential gene expression during acute SIVmac239 infection of RMs. Bar plots of differentially expressed genes relative to day 0 baseline (blood draw prior to SIVmac239 challenge later in day) in **(A)** vaccinated Mamu-B*08+ RMs, unvaccinated Mamu-B*08+ RMs, and unvaccinated Mamu-B*08^–^ RMs, **(B)** unvaccinated Mamu-B*08+ ECs and CPs, and **(C)** vaccinated Mamu-B*08+ ECs and CPs. Red indicates upregulated genes; blue indicates downregulated genes.

To test this hypothesis, we performed functional genomics analysis to define the global transcriptomes of whole blood samples in our 40-animal cohort at multiple timepoints during the first 14 days of SIVmac239 infection: day 0 (prior to infecting challenge to establish baseline levels of gene expression), day 3, day 7, day 10, and day 14. We first performed principal component analysis (PCA) of transcriptome data sets for dimensionality reduction and visualization of technical and biological variation in gene expression and added sex as a model covariate. Though we did not observe differential clustering when comparing ECs and CPs by PCA, data points from each post-infection timepoint formed shifting clusters, as expected (**Supplementary Figure S7**). Differential gene expression (DE) analysis identified significant changes in gene expression profiles relative to a pre-infection baseline timepoint by day 10 post-infection for all experimental groups, independent of vaccination/EC status or MHC class I genotype (**Figure 9** and **Supplementary Data S1**). The kinetics of differential gene expression were slightly delayed in Mamu-B*08^–^ RMs relative to Mamu-B*08+ RMs. At day 10 post-infection, only 347 genes underwent significant changes in expression in unvaccinated Mamu-B*08^–^ RMs, compared to 1,281 and 2,101 genes for vaccinated and unvaccinated Mamu-B*08+ RMs, respectively. At day 14 post-infection, vaccinated Mamu-B*08+ RMs had fewer DE genes (1,129) than unvaccinated Mamu-B*08+ RMs (2,672) and unvaccinated Mamu-B*08^–^ RMs (2,140). Upregulated genes in each group of animals were heavily enriched in innate immune processes, including immunomodulatory genes such as *APOBEC3B*, *IFI27*, *IFI44*, *ISG15*, *ISG20*, *OAS1*, *OAS2*, *RIGI*, *RSAD2*, *STAT1*, *STAT2*, and *TLR7* (**Supplementary Data S1**).

**Figure 10:**
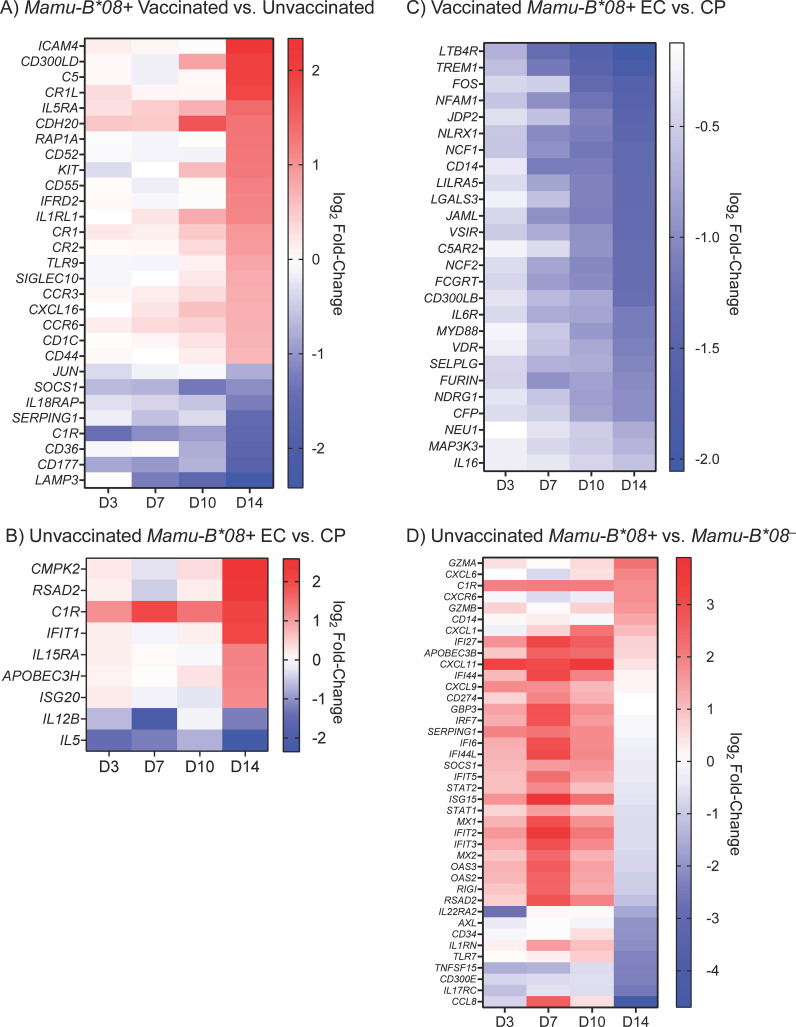
Differentially expressed immunomodulatory genes in vaccinated, unvaccinated, Mamu-B*08+, and Mamu-B*08^–^ RMs during acute SIVmac239 infection. Heat maps depict selected differentially expressed immunomodulatory genes during the first 14 days of SIVmac239 infection in **(A)** vaccinated Mamu-B*08+ RMs and unvaccinated Mamu-B*08+ RMs, **(B)** unvaccinated Mamu-B*08+ ECs and unvaccinated Mamu-B*08+ CPs, **(C)** vaccinated Mamu-B*08+ ECs and vaccinated Mamu-B*08+ CPs, and **(D)** unvaccinated Mamu-B*08+ RMs and unvaccinated Mamu-B*08^–^ RMs. Differential expression criteria for the genes shown in **(A, C)** were a statistically significant adjusted *P*-value (*P* < 0.05) and |log_2_ fold-change| ≥ 1.5 for at least one of the four timepoints shown. Differential expression criteria for the genes shown in **(B, D)** were a statistically significant non-adjusted *P*-value (*P* < 0.05) and |log_2_ fold-change| ≥ 1.5 for at least one of the four timepoints shown. Red indicates upregulated genes; blue indicates downregulated genes.

To determine whether Mamu-B*08+ RMs and RMs that ultimately become ECs exhibit a unique gene expression profile during the acute phase of SIV infection, we performed multiple analyses comparing DE gene subsets for various groupings of the 40 animals in our cohort. Because vaccination enhances the EC phenotype in Mamu-B*08+ RMs, we first compared the global transcriptomic profiles of vaccinated and unvaccinated Mamu-B*08+ RMs, identifying a total of 470 differentially expressed genes, a subset of which encode proteins with well-characterized immunomodulatory functions (**Figure 10A** and **Supplementary Data S2**). The magnitudes of differential expression were greatest at day 14 post-infection. Vaccinees expressed higher levels of certain complement components and receptors (C5, CR1L, CR1, CR2), chemokine receptors and ligands (CCR3, CXCL16, CCR6), cytokine receptors (IL5RA, IL1RL1), and toll-like receptor 9 (TLR9). In contrast, unvaccinated controls expressed higher levels of the immunomodulatory transcription factor Jun, complement component C1R, the anti-inflammatory protein SOCS1, and IL18RAP. DE genes were significantly enriched for innate biological processes including neutrophil aggregation (FDR = 0.0178) and complement regulation (FDR = 0.026) by GO enrichment analysis. Next, to focus solely on “natural” and not vaccine-enhanced elite control in Mamu-B*08+ RMs, we compared the transcriptomic profiles of unvaccinated Mamu-B*08+ RMs that became ECs and CPs. Although we were unable to identify any DE genes using standard significance cutoffs (defined as adjusted *P*-values < 0.05 and and |log_2_ fold-change in gene expression| ≥ 1.5), we identified 170 genes that met differential expression criteria using unadjusted *P-*values (**Supplementary Data S3**). This set of genes included a small number of innate immune and immunomodulatory genes: *RSAD2*, *C1R*, *IFIT1*, *IL15RA*, *APOBEC3H*, and *ISG20* were more highly expressed in ECs, while *IL12B* and *IL5* were more highly expressed in CPs (**Figure 10B**).

We then analyzed the vaccinated Mamu-B*08+ RM cohort to better define the transcriptomic correlates of vaccine-enhanced elite control. A total of 197 genes were differentially expressed in vaccinated ECs (n = 13) and vaccinated CPs (n = 3), many of which encode proteins with immunomodulatory functions (**Supplementary Data S4**). The majority of these genes were significantly downregulated in vaccinated ECs relative to vaccinated CPs, particularly at day 14 post-infection (**Figure 10C**). Downregulated DE genes were significantly enriched for innate immune processes (FDR = 0.0186). Finally, we compared the acute-phase transcriptomic profiles of unvaccinated Mamu-B*08+RMs and unvaccinated Mamu-B*08^–^ RMs to determine whether DE genes are associated with the elite control predisposition of Mamu-B*08+ RMs. This resulted in the identification of only one differentially expressed gene (*AXL*). To further explore the data set, we used non-adjusted *P*-values to identify an additional 385 genes (non-adjusted *P*-value < 0.05) that may differentiate acute-phase immune responses in Mamu-B*08+ RMs and Mamu-B*08^–^ RMs (**Figure 10D** and **Supplementary Data S5**). Expression levels of many genes were upregulated in Mamu-B*08+ RMs as early as day 3 post-infection, including a number of genes encoding innate immune proteins with roles in innate immune activation and direct antiviral activities (APOBEC3B, GBP3, OAS2, OAS3, RIGI, RSAD2), chemokine ligands (CXCL1, CXCL11, CXCL9), complement component C1R, and other IRF-3 target genes and interferon-inducible proteins (IFI27, IFI44, IFI44L, IFIT5, ISG15, IFIT2, IFIT3) (35). By day 14 post-infection, granzymes A and B were upregulated in Mamu-B*08+ RMs, as well as CXCL6 and CXCR6.

## 4 Discussion

Elucidating the mechanistic basis of elite control of HIV/SIV viremia in individuals expressing specific MHC class I allotypes could greatly facilitate the development of novel vaccination and cure strategies that recapitulate the elite control phenotype in the general population, even in the absence of elite control-associated MHC class I allotypes. SIVmac239 infection of RMs provides an excellent model system for studying the elite control phenomenon, particularly in Mamu-B*08+ animals, of which approximately 50% spontaneously control chronic-phase viremia of this highly pathogenic SIV clone. In this study, we established a cohort of 32 SIVmac239-infected Mamu-B*08+ RMs to gain further insight into the immunological mechanisms responsible for the remarkable elite control phenotype exhibited by these animals. Although vaccine-mediated boosting of Mamu-B*08-restricted, EC-associated CTLs specific for immunodominant Vif- and Nef-derived epitopes did not protect against intrarectal SIVmac239 acquisition, vaccinees had significantly lower peak and setpoint viremia than unvaccinated controls and maintained EC status (plasma viral load ≤ 10,000 vRNA copies/mL) for significantly longer periods of time than unvaccinated controls. Analyses of cellular immune responses revealed that peripheral blood frequencies of CTLs mounting responses to the minimal optimal Mamu-B*08-restricted Vif RL8 and Vif RL9 epitopes, but not frequencies of CTLs with TCRs binding the corresponding pMHCI tetramers, correlated with an increased number of intrarectal challenges required to establish SIVmac239 infection. Despite an apparent enhancement of the EC phenotype in vaccinees relative to unvaccinated controls (EC frequencies of 81% and 44%, respectively), we did not identify any statistically significant correlations between the quantity or quality of vaccine-induced CTLs in circulation and setpoint viremia. These results are generally consistent with those of our previous studies, although we did previously identify a statistically significant negative correlation between frequencies of Nef RL10-Mamu-B*08 tetramer^+^ CTLs and viral load at day 14 post-infection (9). Nonetheless, we subsequently found that vaccination of Mamu-B*08+ RMs with Nef alone did not enhance the EC phenotype, likely due to increased selective pressures for mutational escape by CTLs recognizing this single epitope (10). Additionally, in our most recent study employing a similar Vif and Nef vaccination regimen in Mamu-B*08+ RMs, we did not identify statistically significant correlations between vaccine-induced CTL parameters and SIVmac239 acquisition rate (12). We conclude that the elite control predisposition of Mamu-B*08+ RMs and elite control enhancement observed in vaccinated Mamu-B*08+ RMs cannot be fully explained by conventional cellular immunological analyses of MHC class I-restricted, SIV-specific CTLs alone.

To further investigate a possible mechanistic basis for the establishment of the EC phenotype, we performed global transcriptomic analysis of whole blood at acute-phase timepoints for the 32 SIVmac239-infected Mamu-B*08+ RMs in our cohort and an additional eight SIVmac239-infected Mamu-B*08^–^ RMs. All of our differential gene expression comparisons among various groupings of the 40 animals based on MHC class I genotype, vaccination status, and eventual EC/CP status yielded relatively small subsets of differentially expressed immunomodulatory genes. Indeed, one major limitation of this study is the use of non-adjusted *P*-values to compare certain differential gene expression patterns, namely between unvaccinated Mamu-B*08+ ECs and CPs (**Figure 10B**), and between unvaccinated Mamu-B*08+ and Mamu-B*08^–^ RMs (**Figure 10D**). While the more comprehensive dataset exploration afforded using non-adjusted *P*-values provided us with additional insight into important transcriptomic differences between these groups, we must exercise caution in interpreting these findings as *bona fide* without additional studies and a larger sample size. Further experiments should also be performed to confirm our differential gene expression observations at the protein level as well as in different types of leukocytes to identify the cell type(s) responsible for the observed differential gene expression patterns. Indeed, such *in vitro* experimentation would allow us to determine whether the identified immunomodulatory gene subsets are functioning in innate or adaptive capacities, or in crosstalk between the innate and adaptive immune systems, during acute SIVmac239 infection.

The kinetics of differential gene expression appear to be a factor in the determination of EC status. Genes involved in innate immune responses were upregulated more rapidly in unvaccinated Mamu-B*08+ RMs than unvaccinated Mamu-B*08^–^ RMs, and more rapidly in Mamu-B*08+ RMs destined to become ECs than those that become CPs. While many of the specific genes identified in these various analyses differ, most encode proteins involved in similar immunological processes, including the complement system, cytokines/chemokines and their receptors, pattern recognition receptors, and proteins involved in innate immune activation and direct viral control. More rapid and robust innate immune responses during acute infection facilitate suppression of viral replication and spread, and promote the innate/adaptive immune interactions that serve to program and expedite the development of efficacious adaptive immune responses and could explain why the eventual EC/CP phenotype appears to be determined early in infection. Yet a direct association between the kinetics and magnitude of such innate immune responses and the expression of a highly specific MHC class I allotype is unclear.

Vaccinated Mamu-B*08+ RMs exhibited even more rapid immune system activation than unvaccinated Mamu-B*08+ RMs, quickly upregulating complement components, cytokines/chemokines and cytokine/chemokine receptors, IRF-3 target genes, and other interferon-activated genes, including viral restriction factors. Somewhat paradoxically, the primary transcriptomic differences between vaccinated Mamu-B*08+ RMs that became ECs and vaccinated Mamu-B*08+ RMs that became CPs involved a marked downregulation of immunomodulatory genes in ECs relative to CPs by day 14 post-infection. All three of the vaccinated CPs in this study had below-average vaccine-induced CTL frequencies (both by pMHCI tetramer staining and CD107a/ICS assay) relative to the entire cohort of vaccinated animals (n = 16) at the time of the first SIV challenge. Thus, the increased expression of innate immune gene products in vaccinated CPs reflects ongoing innate immune activation, perhaps indicative of suboptimal vaccine-induced CTL responses.

The immunomodulatory genes identified in previous transcriptomic studies of HIV EC patients share many similarities to the differentially expressed gene subsets we identified in our Mamu-B*08+ SIV ECs. A number of innate immune and interferon-stimulated genes are upregulated in HIV ECs. Innate immune and interferon-stimulated genes upregulated in both our Mamu-B*08+ SIV ECs and in the HIV ECs of these previously published datasets include *CMPK2*, *IFI27*, *IFI6*, *IFI44*, *IFI44L*, *SERPING1*, *MX1*, and *OAS3* (**Figures 10B, D**) (36–38). Inconsistent with our acute-phase findings of upregulated inflammatory responses in ECs (e.g. pattern recognition receptors and chemokine ligands) are previous reports of inflammatory response downregulation in ECs, albeit at later timepoints post-infection than in our analyses (37, 38). Thus, the apparent rapid upregulation and resolution of these inflammatory responses in Mamu-B*08+ RMs relative to Mamu-B*08^–^ RMs (**Figure 10D**) may in fact be compatible with the findings of these previous studies.

Indeed, earlier resolution of the pro-inflammatory innate immune responses during hyperacute infection may serve to mitigate disease progression in Mamu-B*08+ RMs. Comparisons of hyperacute-phase whole blood transcriptomic profiles of SIVmac239-infected RMs and SIVsmm-infected sooty mangabeys (SMs) support this idea (39). While SIVmac239 disease progression is rapid in non-EC RMs, with most animals developing AIDS within one year of infection (40), the overwhelming majority of SIVsmm-infected SMs do not develop AIDS despite experiencing relatively high chronic-phase viral loads (41). While SIVsmm-infected SMs do not exhibit a true EC phenotype, their hyperacute-phase immune responses are more rapid, more intense, and resolve more quickly than those of SIVmac239-infected RMs (39). Thus, the kinetics, intensity, and early resolution of hyperacute-phase immune responses in AIDS-refractory SMs relative to AIDS-susceptible RMs are analogous to our observations in EC-predisposed Mamu-B*08+ RMs and CP Mamu-B*08^–^ RMs. More recently, Kazer et al. performed longitudinal single-cell RNA-sequencing analysis of PBMCs from four HIV-infected patients, two of whom became ECs (42). Remarkably, Kazer et al. were able to capture both pre-infection and hyperacute phase timepoints in these patients, providing some insight into the same early immune responses we analyzed in our SIV-infected RM cohort. Both ECs exhibited higher levels of acute-phase CTL proliferation than CPs and possessed a unique subset of proliferating NK cells (42). Interestingly, neither of the ECs expressed the EC-associated HLA-B*27 or HLA-B*57 MHC class I allotypes, but one of the CPs was HLA-B*27+. Thus, these ECs may not be the best comparators for our Mamu-B*08+ RM ECs, where the EC phenotype is much more strongly associated with MHC class I haplotype. Although the biological significance of the observations of Kazer et al. may be limited due to the very small sample size (four patients), future studies could expand upon this dataset and more clearly delineate the hyperacute immune responses that appear to contribute to the EC phenotype.

In this study, we established a large cohort of SIVmac239-infected RMs with a predisposition for MHC class I-associated elite control. While prophylactic vaccination to elicit EC-associated, SIV-specific CTLs clearly enhanced the EC phenotype, our inability to identify meaningful correlations between vaccine-induced CTL parameters in peripheral blood and setpoint viremia implicate additional immunological factors in the determination of EC status. Our transcriptomic analyses of Mamu-B*08+ RMs, both vaccinated and unvaccinated, and Mamu-B*08^–^ RMs during the first two weeks of SIVmac239 infection suggest that numerous innate immune factors, as well as the intensity and duration of pro-inflammatory innate immune responses, differentiate ECs from CPs. Additionally, our identification of unique subsets of differentially expressed immunomodulatory genes in our various RM group comparisons suggests that the precise mechanism of vaccine-enhanced elite control may differ from that of naturally occurring elite control in Mamu-B*08+ RMs. Elucidation of the mechanistic basis for MHC class I-associated elite control of HIV/SIV viremia will ultimately require transcriptomic studies at single-cell resolution during the acute-phase of SIV infection in nonhuman primates. A deeper, more comprehensive understanding of the remarkable immunology of Mamu-B*08+ RMs during SIVmac239 infection could ultimately facilitate the development of novel HIV/AIDS therapeutic strategies recapitulating the EC phenotype, bringing us closer to ending the HIV/AIDS pandemic.

## Data Availability

The raw sequencing data generated in this study have been deposited in the NCBI Gene Expression Omnibus (GEO) under accession number GSE272837.
