# Supplementary material for: Acute-phase innate immune responses in SIVmac239-infected Mamu-B*08+ Indian rhesus macaques may contribute to the establishment of elite control
Source: Front Immunol. 2024 Oct 22;15:1478063. doi: 10.3389/fimmu.2024.1478063 (PMC11534762; doi:10.3389/fimmu.2024.1478063)
Supplement: Supplementary file 1 [file DataSheet1.pdf]

Supplementary Material

1 Supplementary Data

Supplementary Data 1. Differentially expressed genes during acute SIVmac239 infection. Animal groupings are the following: Group 1 – vaccinated *Mamu-B*08*+ RMs, Group 2 – unvaccinated *Mamu-B*08*+ RMs, Group 3 – unvaccinated *Mamu-B*08*$^{-}$ RMs. P_{adj} = adjusted *P*-value.

Supplementary Data 2. Differentially expressed genes in vaccinated and unvaccinated *Mamu-B*08*+ RMs during acute SIVmac239 infection. P_{adj} = adjusted *P*-value.

Supplementary Data 3. Differentially expressed genes in unvaccinated *Mamu-B*08*+ ECs and CPs during acute SIVmac239 infection. *P* = non-adjusted *P*-value.

Supplementary Data 4. Differentially expressed genes in vaccinated *Mamu-B*08*+ ECs and CPs during acute SIVmac239 infection. P_{adj} = adjusted *P*-value.

Supplementary Data 5. Differentially expressed genes in unvaccinated *Mamu-B*08*+ RMs and unvaccinated *Mamu-B*08*$^{-}$ RMs during acute SIVmac239 infection. *P* = non-adjusted *P*-value.

2 Supplementary Figures and Tables

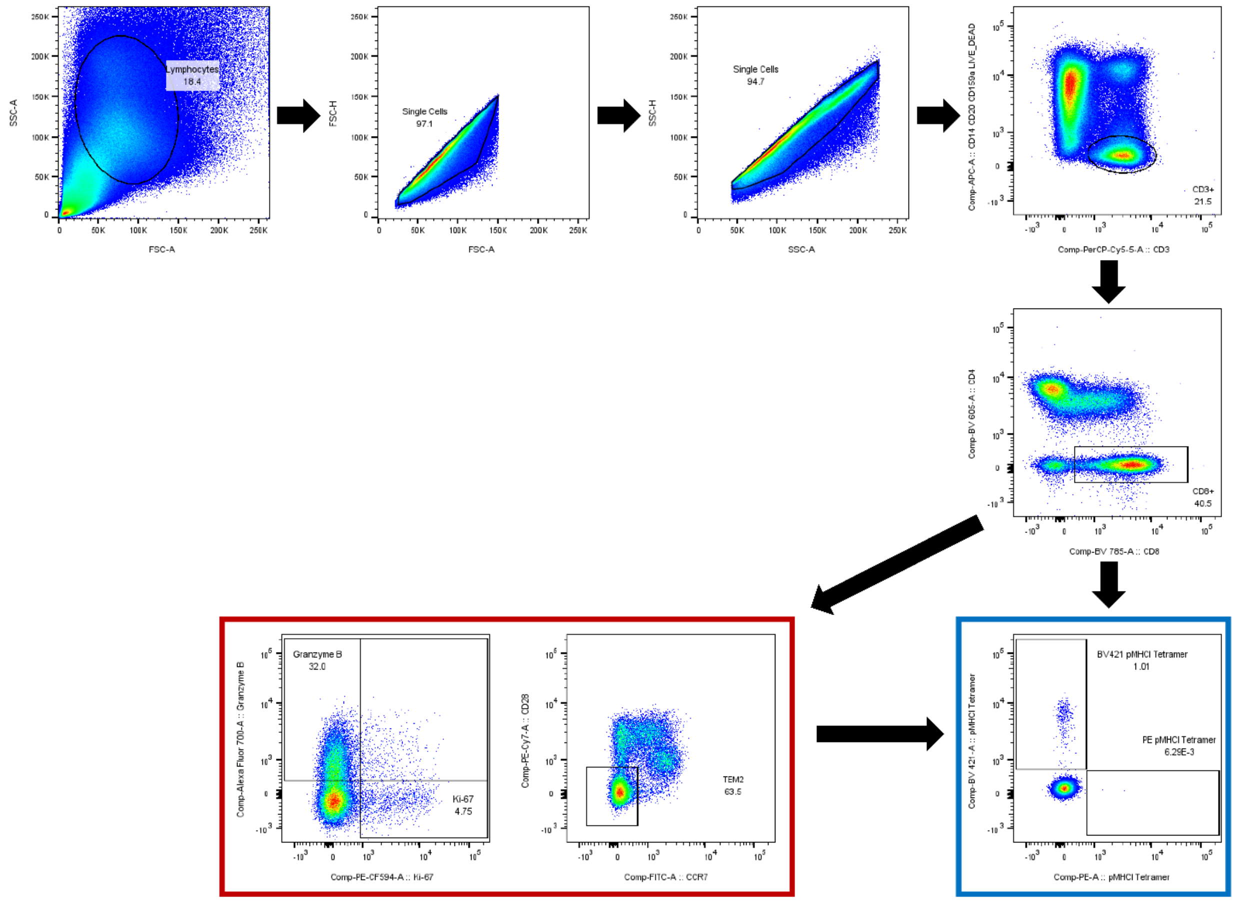

Supplementary Figure 1. Flow cytometry gating strategy for pMHCI tetramer staining and CTL phenotypic characterization. Phenotyping gates (red box) were established based upon all $CD3^+$ $CD8^+$ $CD4^-$ T cells then applied to the tetramer-positive populations of interest, shown in blue. Each staining tube contained two Mamu-B*08 pMHCI tetramers loaded with different peptides and conjugated to different fluorophores (either PE or BV421). This representative pMHCI tetramer stain shows co-staining of vaccinee CTLs with a BV421-conjugated Nef RL10-Mamu-B*08 tetramer and a PE-conjugated Nef RL9b-Mamu-B*08 tetramer.

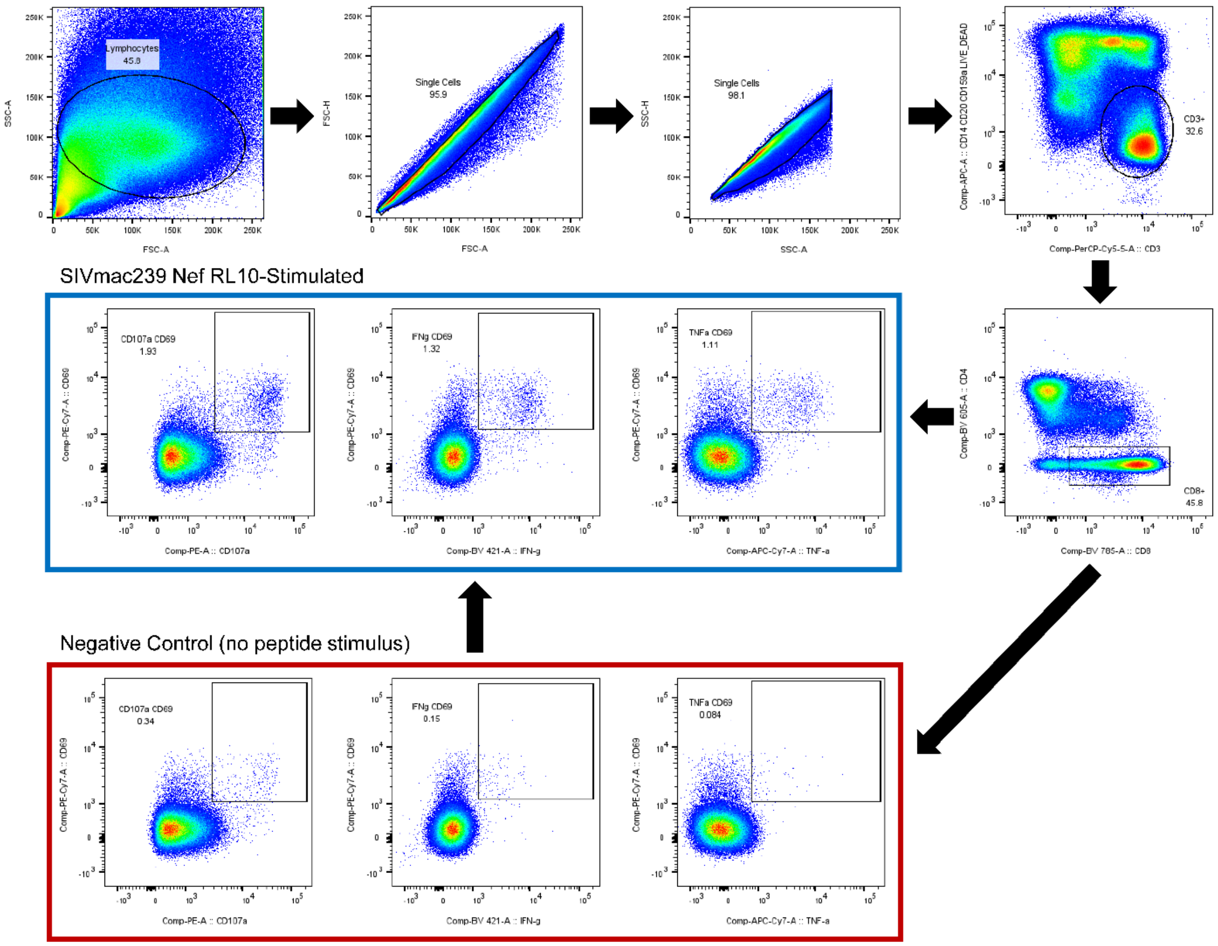

Supplementary Figure 2. Flow cytometry gating strategy for CD107a/ICS assays. Unstimulated PBMCs from each animal were used to establish appropriate gating for CD69 co-expression with each of the three effector function markers (CD107a, IFN-γ, and TNF-α), shown in the red box. These gates were then applied to peptide-stimulated PBMCs (blue box) to determine whether CTL responses to a given peptide exceeded background staining observed in unstimulated PBMCs. Boolean gating (performed in FlowJo) was used to assess all possible combinations of effector functions. Detectable responses were defined as *or* gate frequencies at least twofold greater than the *or* gate frequency for the unstimulated negative control condition. Frequencies of responding CTLs were quantified by subtracting background response frequencies for the unstimulated negative control from the frequencies observed under each stimulation condition.

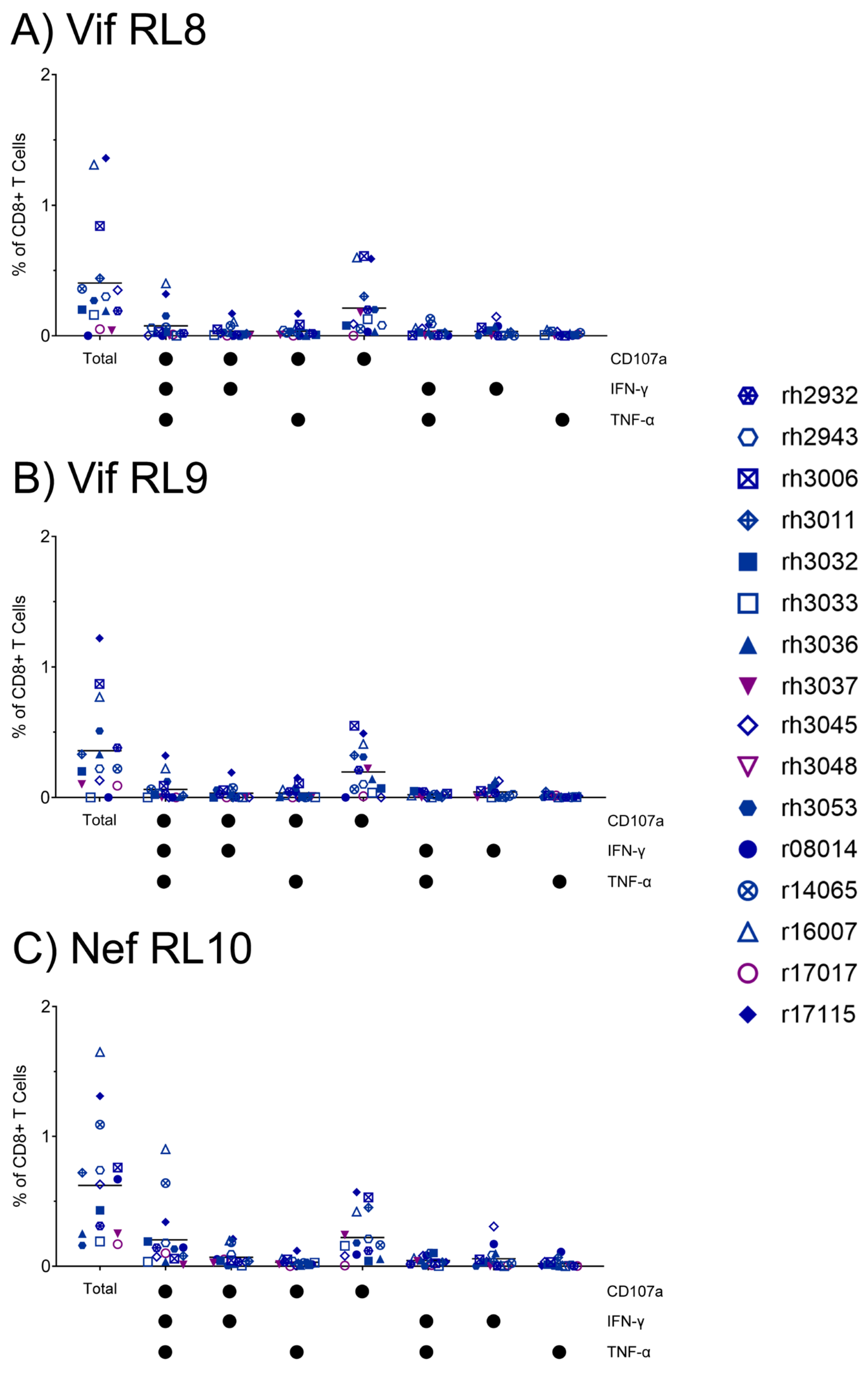

Supplementary Figure 3. Frequencies of CTLs responding to immunodominant Mamu-B*08-restricted Vif- and Nef-derived CTL epitopes at the time of the first SIVmac239 challenge. Plots depict frequencies of CD69$^+$ CTLs staining positive for the indicated combinations of effector function markers in response to stimulation with (A) Vif RL8, (B) Vif RL9, and (C) Nef RL10 in a CD107a/ICS assay. Responding CTLs were defined as CD69$^+$ CD3$^+$ CD8$^+$ CD4$^-$ CD14$^-$ CD20$^-$ CD159a$^-$ lymphocytes staining positive for CD107a, IFN-γ, or TNF-α.

A)

Animal ID	IR SIVmac239 Dose		# of Challenges to Infection
	$TCID_{50}$	vRNA Copies	
rh2756	1,000	1.07×10^8	1
r05035	1,000	1.07×10^8	1
rh2810	100	1.07×10^7	1
rh3000	100	1.07×10^7	1
rh3013	10	1.07×10^6	2
rh2848	10	1.07×10^6	2
rh2995	10	1.07×10^6	7
rh2861	10	1.07×10^6	3

B)

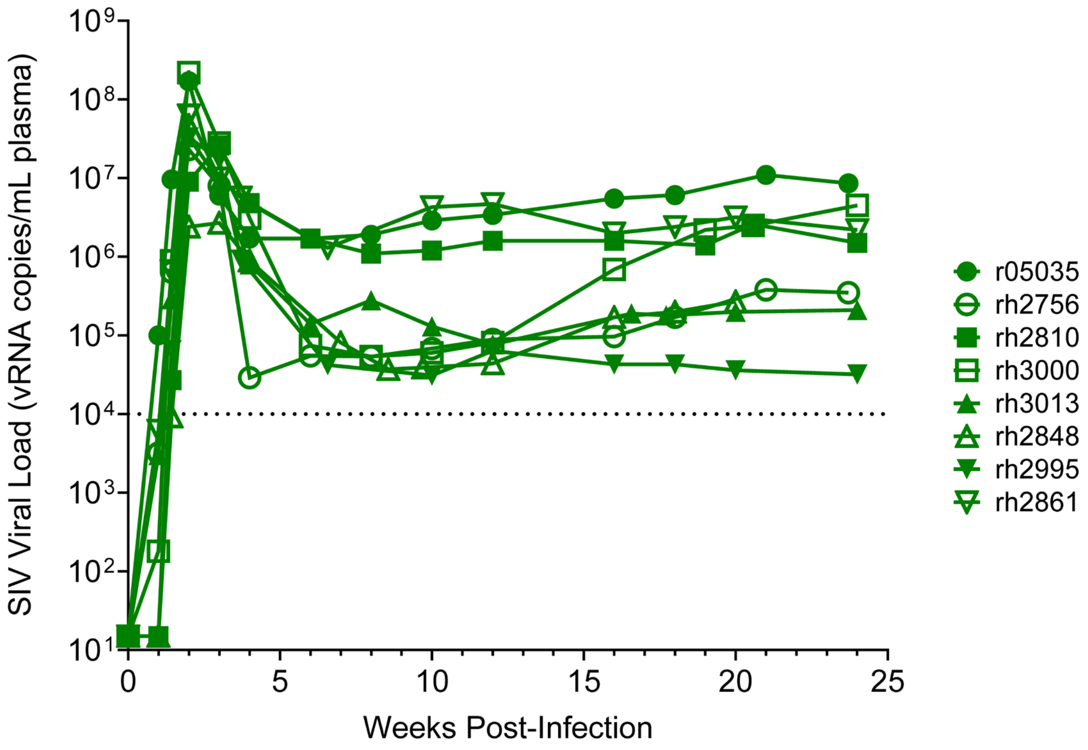

Supplementary Figure 4. SIVmac239 acquisition rates and longitudinal viral loads for unvaccinated *Mamu-B*08*⁻ RMs. RMs were infected by intrarectal challenge with the same clonal rhesus PBMC-passaged SIVmac239 stock used to infect the 32 *Mamu-B*08+* RMs in this study. (A) SIVmac239 intrarectal challenge doses and number of challenges to infection; (B) longitudinal SIVmac239 plasma viral loads. None of the eight RMs in this figure expressed the elite control-associated Mamu-B*08 or Mamu-B*17 MHC class I allotypes (see Table 1).

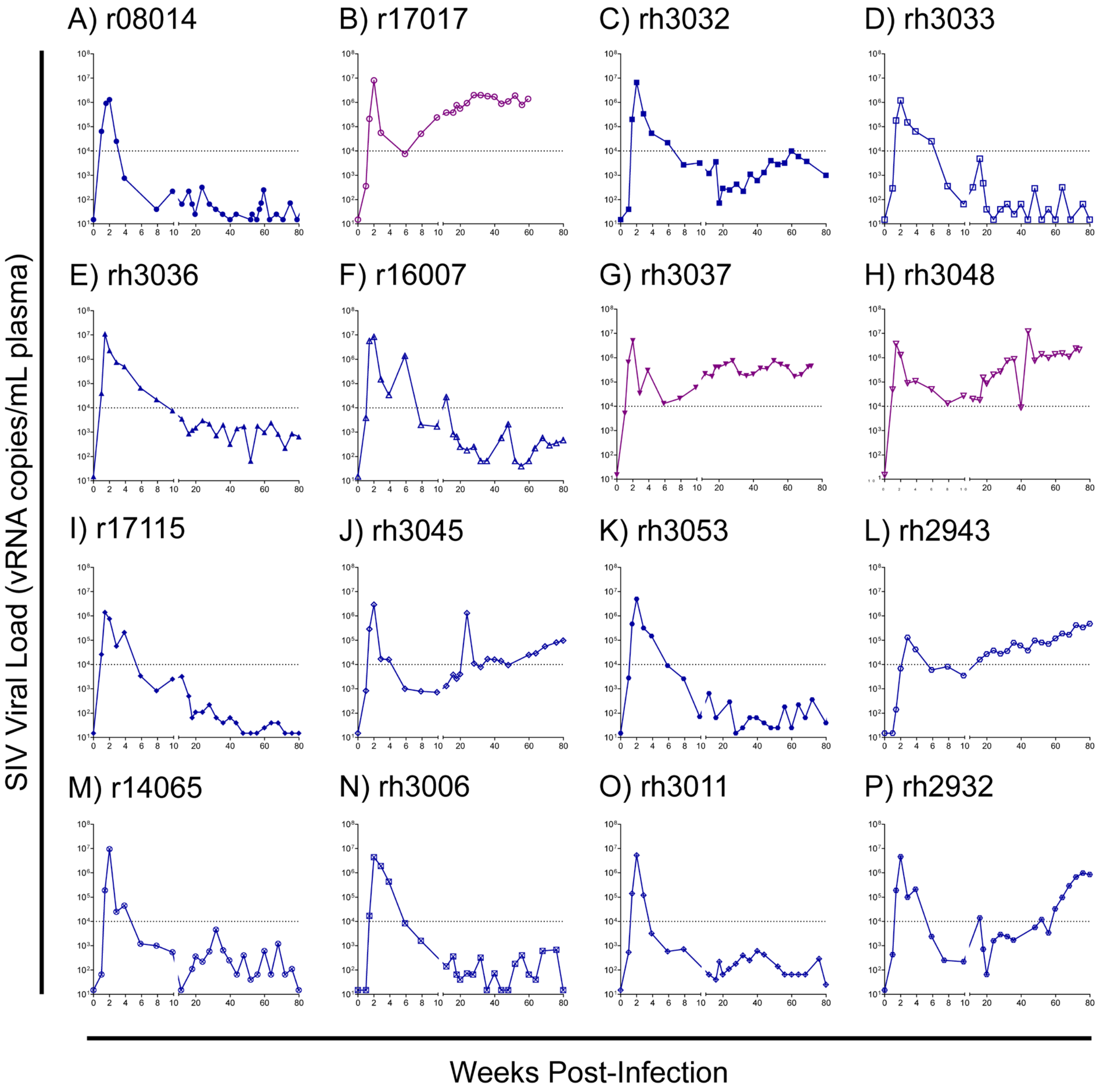

Supplementary Figure 5. SIVmac239 viral load plots for individual vaccinated *Mamu-B*08+* RMs in this study. Longitudinal plasma SIV viral loads for vaccinated *Mamu-B*08+* RMs that became ECs (blue) and CPs (purple).

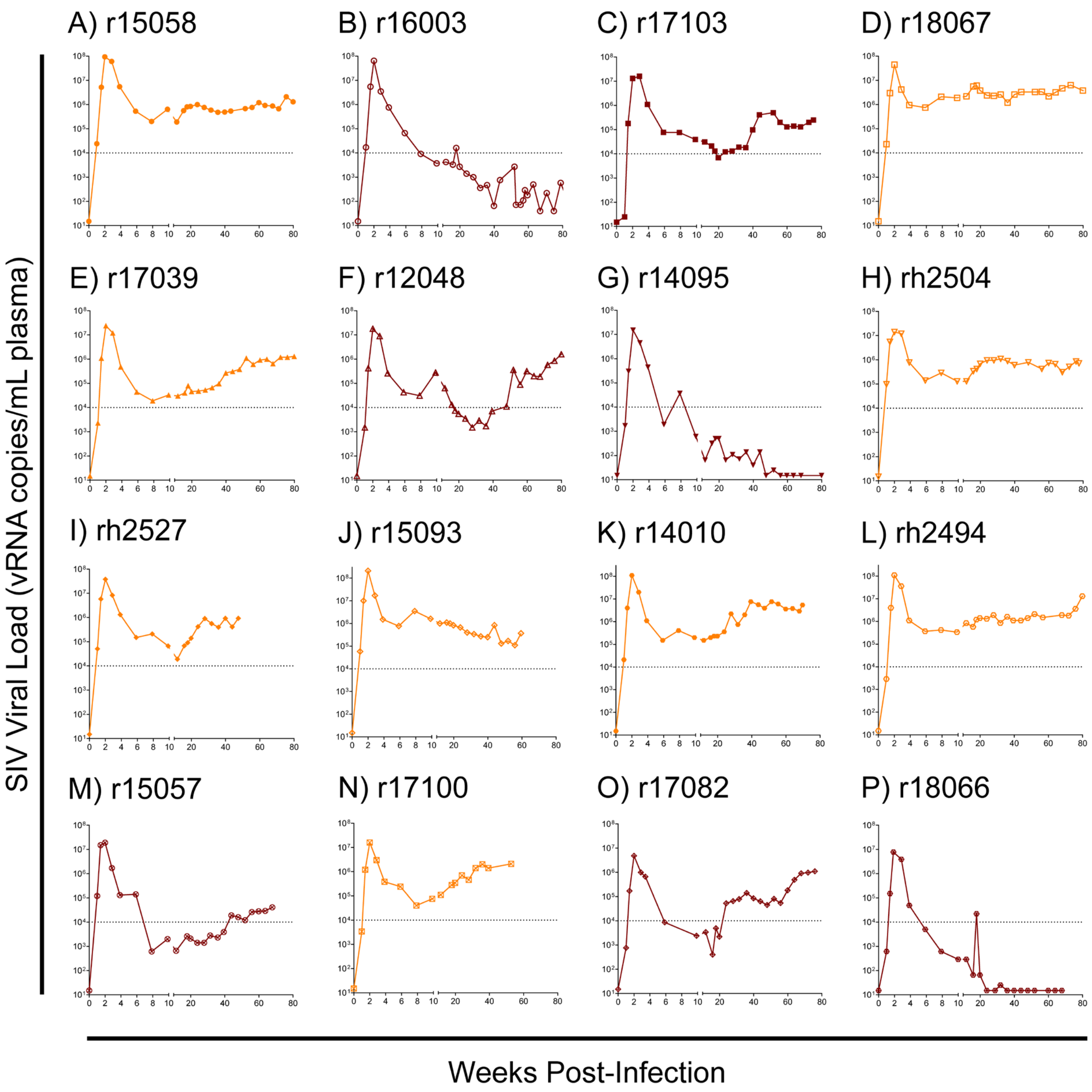

Supplementary Figure 6. SIVmac239 viral load plots for individual unvaccinated *Mamu-B*08*+ RMs in this study. Longitudinal plasma viral loads for unvaccinated *Mamu-B*08*+ RMs that became ECs (red) and CPs (orange).

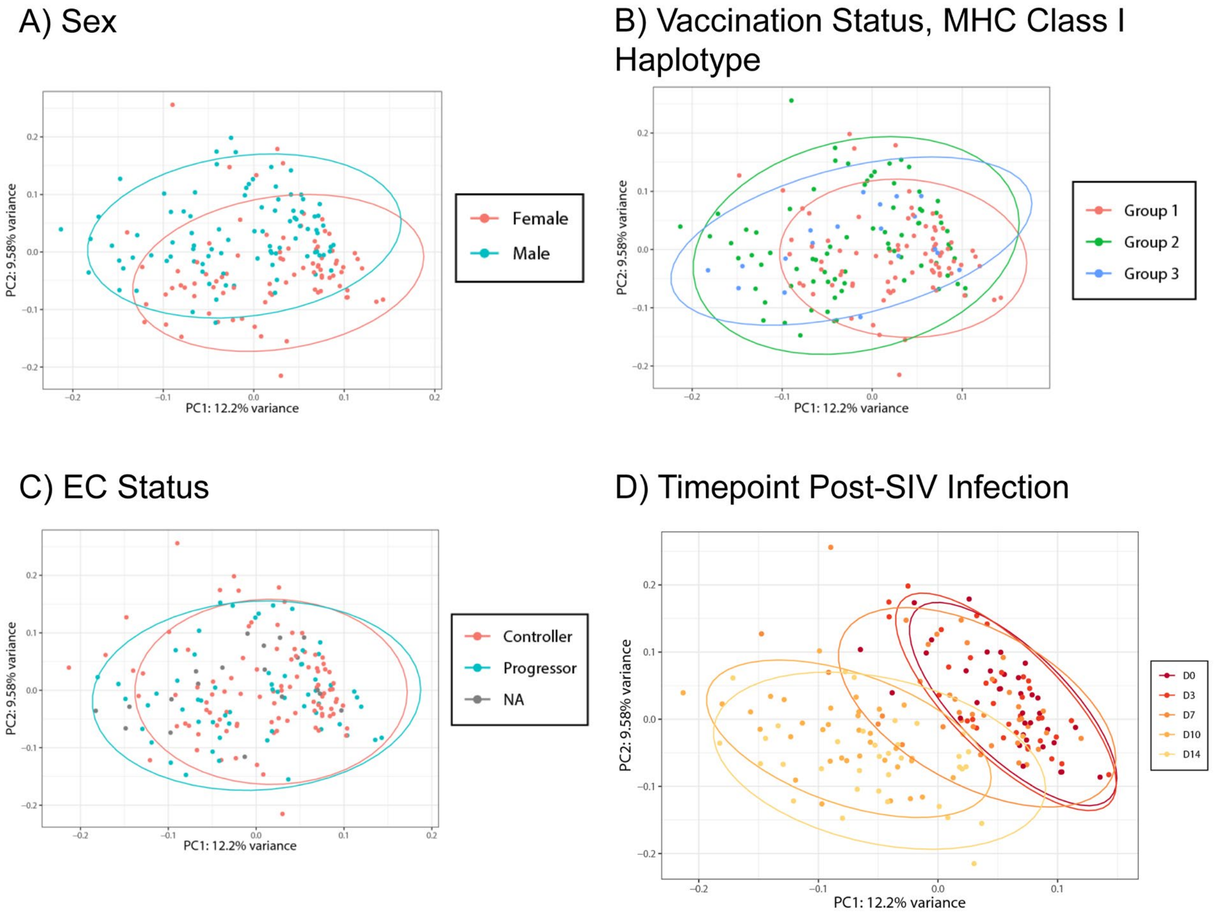

Supplementary Figure 7. RNA-sequencing principal component analysis (PCA). PCA based on (A) RM sex, (B) vaccination status and MHC class I haplotype, (C) EC status, and (D) timepoint post-SIV infection.
